# Mutations in *degP* and *spoT* Genes Mediate Response to Fermentation Stress in Thermally Adapted Strains of Acetic Acid Bacterium *Komagataeibacter medellinensis* NBRC 3288

**DOI:** 10.3389/fmicb.2022.802010

**Published:** 2022-05-12

**Authors:** Naoya Kataoka, Minenosuke Matsutani, Nami Matsumoto, Misuzu Oda, Yuki Mizumachi, Kohei Ito, Shuhei Tanaka, Yu Kanesaki, Toshiharu Yakushi, Kazunobu Matsushita

**Affiliations:** ^1^Department of Biological Chemistry, Faculty of Agriculture, Yamaguchi University, Yamaguchi, Japan; ^2^Graduate School of Sciences and Technology for Innovation, Yamaguchi University, Yamaguchi, Japan; ^3^Research Center for Thermotolerant Microbial Resources, Yamaguchi University, Yamaguchi, Japan; ^4^NODAI Genome Research Center, Tokyo University of Agriculture, Tokyo, Japan; ^5^Research Institute of Green Science and Technology, Shizuoka University, Shizuoka, Japan

**Keywords:** DegP, SpoT, acetic acid fermentation, thermotolerance, experimental evolution, cell length, membrane vesicle

## Abstract

An acetic acid bacterium, *Komagataeibacter medellinensis* NBRC 3288, was adapted to higher growth temperatures through an experimental evolution approach in acetic acid fermentation conditions, in which the cells grew under high concentrations of ethanol and acetic acid. The thermally adapted strains were shown to exhibit significantly increased growth and fermentation ability, compared to the wild strain, at higher temperatures. Although the wild cells were largely elongated and exhibited a rough cell surface, the adapted strains repressed the elongation and exhibited a smaller cell size and a smoother cell surface than the wild strain. Among the adapted strains, the ITO-1 strain isolated during the initial rounds of adaptation was shown to have three indel mutations in the genes *gyrB*, *degP*, and *spoT*. Among these, two dispensable genes, *degP* and *spoT*, were further examined in this study. Rough cell surface morphology related to *degP* mutation suggested that membrane vesicle-like structures were increased on the cell surface of the wild-type strain but repressed in the ITO-1 strain under high-temperature acetic acid fermentation conditions. The Δ*degP* strain could not grow at higher temperatures and accumulated a large amount of membrane vesicles in the culture supernatant when grown even at 30°C, suggesting that the *degP* mutation is involved in cell surface stability. As the *spoT* gene of ITO-1 lost a 3′-end of 424 bp, which includes one (Act-4) of the possible two regulatory domains (TGS and Act-4), two *spoT* mutant strains were created: one (ΔTGSAct) with a drug cassette in between the 5′-half catalytic domain and 3′-half regulatory domains of the gene, and the other (ΔAct-4) in between TGS and Act-4 domains of the regulatory domain. These *spoT* mutants exhibited different growth responses; ΔTGSAct grew better in both the fermentation and non-fermentation conditions, whereas ΔAct-4 did only under fermentation conditions, such as ITO-1 at higher temperatures. We suggest that cell elongation and/or cell size are largely related to these *spoT* mutations, which may be involved in fermentation stress and thermotolerance.

## Introduction

Acetic acid bacteria are strictly aerobic Gram-negative bacteria that are known for their ability to oxidize various alcohols, sugars, and sugar alcohols. Vinegar is industrially produced, employing the oxidative fermentation ability of acetic acid bacteria, especially *Acetobacter* and *Komagataeibacter*, and is widely used in many food products. In particular, *Komagataeibacter* sp. has been used to produce high concentrations of acetic acid because of its high acetic acid tolerance. During the fermentation process, acetic acid bacteria are exposed to severe stress by the acetic acid produced and the substrate ethanol. Furthermore, acetic acid fermentation is hindered by fermentation heat, which may worsen the stressor’s negative effect, and thus requires strict temperature control. Thus, developing thermotolerant strains of *Komagataeibacter* sp. would be useful to enable efficient high-temperature and high-acidity fermentation.

Experimental evolution is one of the means of obtaining thermotolerant microorganisms. Thermal adaptation has been previously achieved in various microorganisms, including *Escherichia coli* (Rudolph et al., 2010; Rodriguez-Verdugo et al., 2014), *Saccharomyces cerevisiae* (Caspeta et al., 2014), *Acetobacter pasteurianus* (Azuma et al., 2009; Matsutani et al., 2013; Matsumoto et al., 2020), *Komagataeibacter oboediens* (Taweecheep et al., 2019), and *Gluconobacter frateurii* (Hattori et al., 2012; Matsumoto et al., 2018). Furthermore, the mechanism associated with thermotolerance was also analyzed using these strains. However, genomic mutations due to thermal adaptation vary from strain to strain in terms of the mode of gene mutation and the expected functions of the mutated genes. Although highly diverse, the mutated genes have been identified to be related to cell surface functions (peptidoglycan synthesis, outer membrane protein, and lipopolysaccharide synthesis), transporters for ions or amino acids, and two-component signal transduction systems (Matsushita et al., 2016). In addition, the “thermotolerant” genes indispensable for growth at higher temperatures have been examined in the acetic acid bacterium *Acetobacter tropicalis* (Soemphol et al., 2011), *E. coli* (Murata et al., 2011), and *Zymomonas mobilis* (Charoensuk et al., 2017), where, based on gene-disruption-dependent thermosensitive mutant selection, the genes related to membrane stabilization, transport, protein quality control, and cell division have been commonly identified (Charoensuk et al., 2017). In *A. tropicalis* SKU1100, 4 among the 32 transposon-induced thermosensitive mutant strains were identified to be defective in the ATPR_1619 gene encoding serine protease DegP (Soemphol et al., 2011), indicating that DegP is indispensable for growth at temperatures higher than the optimum. This periplasmic protease has been also identified as a “thermotolerant gene” in *E. coli* (Murata et al., 2011) and *Z. mobilis* (Charoensuk et al., 2017), as well as in the plant *Arabidopsis* (Charng et al., 2007), and shown to be related to ethanol tolerance in addition to thermotolerance in *A. tropicalis* (Soemphol et al., 2011) and *Z. mobilis* (Charoensuk et al., 2017).

DegP is known to act as a chaperone at low temperatures but switches to a peptidase (heat shock protein) at higher temperatures (Spiess et al., 1999). Thus, abnormal proteins produced at elevated temperatures can be degraded by DegP, which may recognize improperly folded or denatured proteins that accumulate in the inner membrane and/or periplasmic space (Strauch et al., 1989; Lipinska et al., 1990). Defects in DegP function have been shown to generate a membrane hyper-vesiculation phenotype in several bacteria (McBroom et al., 2006; McBroom and Kuehn, 2007; Schwechheimer et al., 2013).

While the RNA polymerase sigma factor has been listed as a thermotolerant gene in *A. tropicalis* in a previous study (Soemphol et al., 2011), the RNA polymerase RpoH and its- b-subunit (RpoB) were also found to be mutated during thermal adaptation of *K. oboediens* (Taweecheep et al., 2019) and *A. pasteurianus* K-1034 (Matsumoto et al., 2021), respectively. In the latter case, the *spoT* gene, which has been shown to interact with RNA polymerase, was also mutated. The *spoT* mutation was also detected in thermally adapted *E. coli* W3110 (Kosaka et al., 2019), with an associated reduction in the amounts of RNA and DNA. SpoT or RelA (RSH family) catalyzes both the synthesis and degradation of guanosine 3′-diphosphate 5′-diphosphate (ppGpp). Disruption of RelA, an additional ppGpp synthetase to SpoT, in *E. coli* K-12 (Yang and Ishiguro, 2003) has been shown to induce thermosensitivity, which was further stimulated by the additional disruption of SpoT but suppressed by certain mutations of RpoB. Thus, SpoT and RNA polymerase working together are expected to play important roles in developing thermotolerance.

The alarmone (p)ppGpp is a mediator of stringent response that coordinates a variety of cellular activities in response to changes in nutritional conditions or environmental stresses. The alarmone is synthesized and degraded by SpoT or RelA (a long-RSH enzyme), which is composed of an N-terminal catalytic domain and a C-terminal regulatory domain (CTD) thought to be involved in the regulation of the catalytic domain (Irving and Corrigan, 2018). The loss of CTD has been shown to increase ppGpp synthetase activity and decrease hydrolase activity (Mechold et al., 2002), while the overexpression of the CTD reduced the ppGpp levels by disturbing oligomerization (Gropp et al., 2001). Furthermore, a number of studies have shown that ppGpp accumulation is related to cell size reduction (Schreiber et al., 1995; Stott et al., 2015; Vadia et al., 2017).

In this study, to obtain a robust strain able to tolerate several fermentation stresses, such as temperature or acetic acid, *Komagataeibacter medellinensis* NBRC3288 was adapted to higher growth temperatures using an experimental evolution approach in the presence of acetic acid and ethanol (fermentation condition). Three adapted strains, ITO-1, ITO-2, and ITO-3, were obtained from NBRC3288 through continuously repeated cultivations at the early growth phase at 34°C, 34.5°C, and 35°C, respectively. The resultant thermally adapted strains were shown to exhibit considerably increased growth and fermentation ability at higher temperatures, with a trade-off of reduced growth than the wild-type strain under non-fermentation conditions. Furthermore, phenotypic observation indicated that the wild-type strain, but not the adapted strains, was elongated and exhibited an irregular cell surface under stressful high-temperature fermentation conditions. Genome mapping analysis of ITO-3 against the wild-type genome revealed a total of eight mutations in the ITO-3 strain. Among the adapted strains, ITO-1 adapted at the initial stage was the focus of this study because it has three unique indel mutations in the genes of *gyrB* (GLX_00040), *degP* (GLX_19020), and *spoT* (GLX_25720). Among the three mutated genes in ITO-1, *spoT* and *degP* were further examined in this study. The results indicate that the *degP* and *spoT* mutations may be involved in the increased thermotolerance and/or acetic acid fermentation abilities of the ITO-1 strain through their influence on cell surface stability and cell division or cell size regulation, respectively.

## Materials and Methods

### Bacterial Strains and Culture Conditions

*Komagataeibacter medellinensis* NBRC3288 was used as the parental strain and as a starting strain for thermal adaptation. *Acetobacter pasteurianus* SKU1108 (Chinnawirotpisan et al., 2003) and its thermally adapted strain, TH-3 (Matsutani et al., 2013), were also used for comparison with the strains obtained in this study.

The strains (colonies from potato agar plates) were first inoculated in a 5-ml potato medium (20-g glycerol, 5-g glucose, 10-g yeast extract [Oriental Yeast, Tokyo, Japan], 10-g polypeptone [Nihon Pharmaceuticals Co. Ltd., Osaka, Japan], and 100-ml potato extract [Matsutani et al., 2013] per 1 L water) and cultivated at 30°C and 120 rpm for 24–36 h until the turbidity of the culture reached 150–200 Klett units. Then, 1% (v/v) of the pre-culture was inoculated into a YPG medium (5-g yeast extract, 5-g polypeptone, and 5-g glycerol per 1 L water), or the YPG medium containing 1% acetic acid and 3% ethanol (YPGAE medium). Culturing was done in a 100-ml medium in a 500-ml Erlenmeyer flask or a baffled flask at different temperatures and 200 rpm shaking. Bacterial growth was measured using a Klett-Summerson photoelectric colorimeter. The acidity of the culture medium at different growth phases was measured by alkali-titration of 1-ml culture with.8 N NaOH using 10 μL of 0.025% phenolphthalein (transition pH range: 8.3–10) as a pH indicator. For the cultivation of *A. pasteurianus* strains, the YPGDE medium (the YPG medium containing.5% glucose and 4% ethanol) was used.

### Thermal Adaptation of *Komagataeibacter medellinensis* Under Acetic Acid Fermentation Conditions at Higher Temperatures

Thermal adaptation of *K. medellinensis* NBRC 3288 was performed in flask culture (Erlenmeyer flask) under fermentation conditions in which the cells grew in the YPGAE medium. This culture condition was chosen as it facilitated high acetic acid production by this strain. First, repeated cultivation was performed at 34°C, with each inoculation being performed at an early log phase in which the culture acidity was between 1.5 and 2% (w/v) (see Supplementary Figure 1). The first cultivation had almost no lag phase, whereas the second and later rounds exhibited long lag phases and low acetic acid production. However, repeated cultivation gradually increased the growth and acidity (Supplementary Figure 1). When almost no lag phase for acetic acid production was detected or acidity higher than the first cultivation was observed, repeated cultivation was stopped and the culture medium was spread on potato and YPGAE agar plates. After cultivation at 30°C, two and three colonies were isolated from potato and the YPGAE plates, respectively, and their growth was compared in a 100-ml YPGAE medium. The smallest colony obtained from the YPGAE plate exhibited better growth and acetic acid production than the other strains at 34°C. This strain, named ITO-1, was used in the next step. The next adaptation cycle was performed with ITO-1 at 34.5°C with 15 repeated cultivations. A single colony named ITO-2, which showed better growth and acetic acid production, was isolated by spreading on YPGAE agar and evaluating its growth in the 100-ml YPGAE medium at 34.5°C. Finally, the ITO-2 strain was adapted at 35°C in the YPGAE medium. At 35°C, a longer lag phase appeared during the early repeated cultivation. However, after 20 repetitions, growth and acetic acid production increased, and a single colony (ITO-3) exhibiting better growth and acetic acid production was identified from the YPGAE agar plate. Because the thermal adaptation at 35°C required a considerably longer time (over 80 days) than the cases of 34°C (23 days) or 34.5°C (35 days), 35°C was judged as the temperature limit for adaptation.

### Construction of Plasmids and Recombinant Strains

The bacterial strains and plasmids used in this study are listed in Supplementary Table 1, and the primers used for PCR are listed in Supplementary Table 2. A putative promoter region of the *adhAB* genes, which encode two subunits of the pyrroloquinoline quinone-dependent alcohol dehydrogenase, was amplified from the genomic DNA of *K. medellinensis* NBRC 3288 with the primers 3288-adhpro-Sac (+) and 3288-adhpro-Xho (−). The *degP* gene (GLX_19020) from NBRC 3288 or ITO-1 strains was amplified from the genomic DNA of *K. medellinensis* NBRC 3288 or ITO-1 with the primers 3288-DegP-PstXho-F and 3288-DegP-HinXba-R. Three fragments, an *adhAB*-gene-promoter construct digested with *Sac*I and *Xho*I, a *degP* gene from NBRC 3288 or ITO-1 digested with *Xho*I and *Xba*I, and the shuttle plasmid pMV24 (Fukaya et al., 1989) digested with *Sac*I and *Xba*I, were ligated to yield pP*degP*^WT^ and pP*degP*^ITO–1^, respectively.

A recombination plasmid was constructed to generate the Δ*degP* strain of *K. medellinensis* NBRC 3288 by inserting the tetracycline resistance (Tc^R^) cassette from pKRP12 (Reece and Phillips, 1995). The *degP* gene was first amplified by PCR from the genomic DNA of *K. medellinensis* NBRC 3288 as described above. The PCR product was digested with *Hin*dIII and cloned into pT7blue cut with *Eco*RV and *Hin*dIII, yielding pT*degP*. Then, a Tc^R^ fragment prepared from pKRP12 by cutting with *Sal*I was ligated with pT*degP* digested with *Sal*I, yielding pΔ*degP*.

ITO-1 has a deletion in the C-terminal regulatory domain in the SpoT protein, which has two sub-domains, TGS and Act-4 (see Figure 3A). In this study, we constructed two mutant strains: one, ΔTGSAct, is a whole regulatory domain deletion mutant, and the other, ΔAct-4, is a mutant missing only the Act-4 domain. The recombination plasmid to generate the ΔTGSAct strain of *K. medellinensis* NBRC 3288 was constructed in the same manner as described above. The *spoT* gene (GLX_25720) was first amplified by PCR from the genomic DNA of *K. medellinensis* NBRC 3288 with the primers 3288-GTPppk-Sal-F and 3288-GTPppk-Xba-R. The PCR product was cloned into a pT7blue cut with *Eco*RV, yielding pT*spoT*. Then, a Tc^R^ fragment prepared from pKRP12 by cutting with *Sal*I was ligated with pT*spoT* digested with *Xho*I, yielding pΔTGSAct.

**FIGURE 1:**
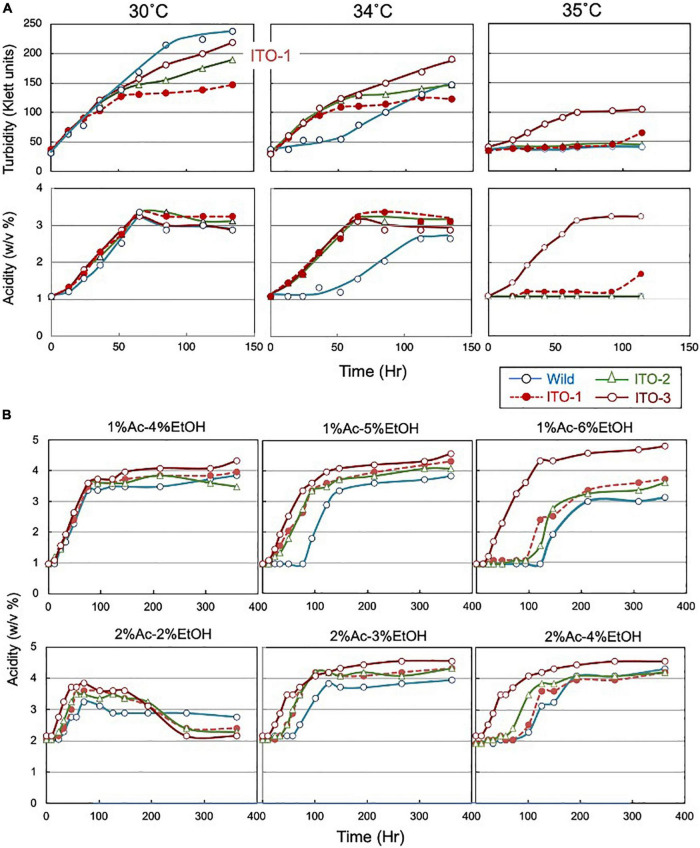
Comparison of growth and acetic acid production of *K. medellinensis* NBRC 3288 and the thermally adapted strains under acetic acid fermentation conditions. **(A)** NBRC 3288 (wild-type strain) and the adapted strains, ITO-1, ITO-2, and ITO-3, were cultivated in the 100-ml of the YPGAE medium in a 500-ml Erlenmeyer flask at 30, 34, and 35°C, with rotary shaking at 200 rpm. Growth (turbidity) and acetic acid concentration (acidity) were measured as described in Materials and methods. This experiment was repeated more than three times, one typical result of which is shown. **(B)** The same strains as **(A)** were cultivated at 30°C in the YPG medium containing different concentrations of acetic acid (Ac) and ethanol (EtOH). In total, 1% Ac-4% EtOH, 1% Ac-5% EtOH, and 1% Ac-6% EtOH show the acetic acid production (acidity) in the YPG medium containing ethanol (4, 5, and 6%, respectively) in the presence of 1% acetic acid, and 2% Ac-2% EtOH, 2% Ac-3% EtOH, and 2% Ac-4% EtOH show the acetic acid production in the YPG medium containing ethanol (2, 3, and 4%, respectively) in the presence of 2% acetic acid.

**FIGURE 2:**
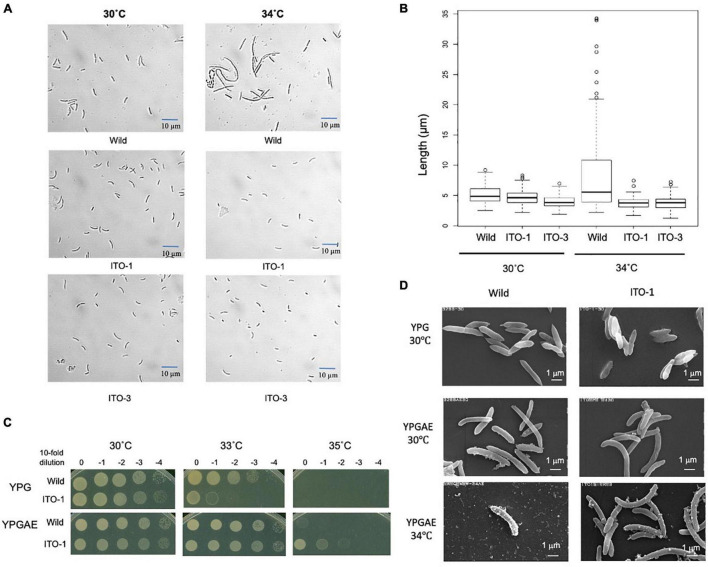
Comparison of cell size and cell surface morphology among NBRC3288, and the adapted strains grown at 30°C and higher temperatures. **(A)** NBRC 3288 (wild-type strain) and the adapted strains, ITO-1 and ITO-3, were cultivated in the 100-ml YPGAE medium in a 500-ml Erlenmeyer flask at 30°C and 34°C with 200-rpm shaking till the early log phase. Cell images were taken under a microscope as described in section “Materials and Methods.” **(B)** The box plot shows the cell sizes of wild-type, ITO-1, and ITO-3 strains grown, as in **(A)**, at 30 and 34°C. The box plots were shown by using PhotoRuler, as described in Materials and methods; median values of the cell lengths in the wild-type, ITO-1, and ITO-3 strains were 4.8, 4.6, and 3.8 μm with the cells grown at 30°C, and 5.5, 3.8, and 3.8 μm with those at 34°C, respectively. **(C)** Dot-spot growth comparison was performed, as described in Materials and methods, between wild-type and ITO-1 strains on YPG and YPGAE media at 30, 33, and 35°C. **(D)** Scanning electron microscopic cell surface morphology of NBRC3288 and ITO-1 strains grown on YPG and YPGAE media at 30 and 34°C.

**FIGURE 3:**
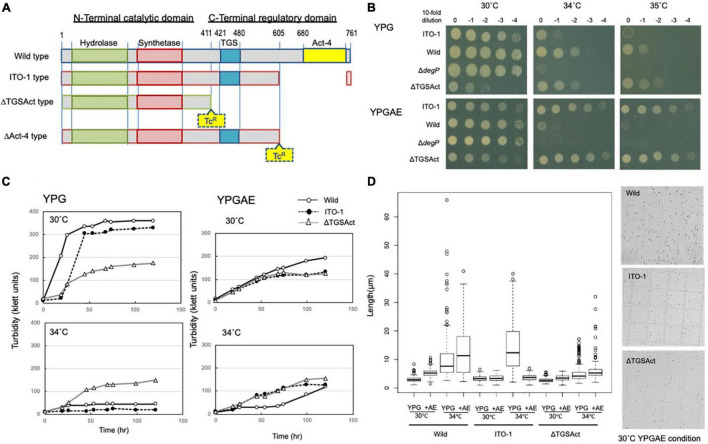
Cell growth and cell length of SpoT mutant strain, ΔTGSAct. **(A)** The *spoT* gene (GLX_25720) domain motif of wild-type and ITO-1 strains, as well as artificial spoT mutant strains, ΔTGSAct, and ΔAct-4. The N-terminal catalytic domain contains metal-dependent hydrolase and synthetase domains, which are thought to constitute a bifunctional enzyme catalyzing (p) ppGpp synthesis, while the C-terminal domain (CTD) is thought to have a TGS domain, which is suggested to be a ligand-binding, regulatory domain, and an Act-4 domain, which may bind to amino acids and regulate associated enzymes. ΔTGSAct and ΔAct-4 were prepared by inserting the Tc^R^ cassette (Tc), as described in section “Materials and Methods.” **(B)** NBRC 3288 (wild-type), ITO-1, Δ*degP*, and ΔTGSAct strains were cultivated in the potato medium containing appropriate antibiotics (25-mg/ml tetracycline for Δ*degP*, and 10-mg/ml ampicillin for ΔTGSAct), and dot-spotted, as described in section “Materials and Methods,” on YPG or YPGAE agar plates, and then incubated at 30, 34, and 35°C. **(C)** NBRC 3288 (wild-type), ITO-1, and ΔTGSAct strains were cultivated in 100-ml of the YPG or YPGAE medium in a 500-ml Erlenmeyer flask at 30 and 34°C. Typical results are shown here from at least three independent cultures of each strain. **(D)** Cell length was measured in wild-type, ITO-1, and ΔTGSAct strains grown on YPG or YPGAE (+AE) at 30 and 34°C. Box plots are shown by using PhotoRuler, as described in Materials and methods, and the median values of each cell grown in YPG were 2.8 (30°C) and 7.7 μm (34°C) in wild-type, 3.2 (30°C) and 12.4 μm (34°C) in ITO-1, and 2.6 (30°C) and 4.2 μm (34°C) in ΔTGSAct, and those of each cell grown in YPGAE were 5.3 (30°C) and 11.6 μm (34°C) in wild-type, 3.5 (30°C) and 3.3 μm (34°C) in ITO-1, and 3.6 (30°C) and 5.3 μm (34°C) in ΔTGSAct.

For construction of the plasmid to generate the ΔAct-4 strain of *K. medellinensis* NBRC 3288, two fragments (N- and C-terminal sequences of the *spoT* gene) were first amplified by PCR from the genomic DNA of *K. medellinensis* NBRC 3288 with the primers 3288-ITO-spoT-5 (+) and 3288-ITO-spoT-fsn-5 (−), and 3288-ITO-spoT-fsn-5 (+) and 3288-ITO-spoT-3 (−), respectively. Three fragments: N- and C-terminal fragments of the *spoT* gene, and the Tc^R^ cassette, were combined by overlap extension PCR using the primers 3288-ITO-spoT-5 (+) and 3288-ITO-spoT-3 (−). The resulting PCR product was cloned into pT7Blue cut with *Eco*RV, yielding pΔAct-4. *K. medellinensis* was transformed with these plasmids by electroporation, according to the manufacturer’s instructions (Bio-Rad, Hercules, CA, United States).

The markerless deletion system based on pKOS6b was used to generate the ΔApAct strain of *A. pasteurianus* SKU1108 (Kostner et al., 2013). To construct the plasmid, two fragments [N- and C-terminal sequences of the *spoT* gene (APT_00593)] were first amplified by PCR from the genomic DNA of *A. pasteurianus* SKU1108 with the primers, 1108-APT0593RI (+) and 1108-APT0593-TGA-Sac (−), and 1108-APT0593-Xba (−) and 1108-APT0593-Sac (+), respectively. Three fragments: an N-terminal fragment cut with *Eco*RI and *Sac*I, a C-terminal fragment cut with *Xba*I and *Sac*I, and pKOS6b cut with *Eco*RI and *Xba*I, were ligated to yield pΔApAct. *A. pasteurianus* was transformed by triparental conjugation (Figurski and Helinski, 1979). Colonies grown on the YPGD medium containing 50 μg/ml kanamycin were isolated as the first recombinant strains and were confirmed by PCR using primers 1108-APT0593RI (+) and 1108-APT0593-Xba (−) to have a wild-type *spoT* DNA band (2 kbp) or a disrupted *spoT* DNA band (1.6 kbp). Next, the first recombinant strains were spread on a plate of the YPGD medium, containing 60 μg/ml 5-fluorocytosine, which was used as a negative selection marker because pKOS6b contains the *codAB* genes (Kostner et al., 2013). For colony isolation, several colonies grown on this plate were confirmed not to grow on a plate of the YPGD medium containing 50-μg/ml kanamycin. Finally, by confirmation of the disrupted *spoT* DNA band (1.6 kbp), the SKU1108 ΔApAct strain was obtained.

### Preparation of Membrane Vesicles

Cells were cultivated in 100 ml of the YPGAE medium in a 500-ml baffled flask at 30°C until the early log phase (Acidity of 1.5∼2%) or the late log to the stationary phase (Acidity of 3.5∼4%). The cell cultures were centrifuged at 10,000 × *g* for 15 min to obtain the supernatants, which were further centrifuged at 133,500 × *g* for 90 min to obtain crude membrane vesicles. The vesicle pellets were resuspended or homogenized with less than 1 ml of a 50-mM potassium phosphate (KPi) buffer (pH 6.5), containing 5-mM MgSO_4_. The suspensions were centrifuged two times at 6,000 × *g* for 2 min to remove debris. Finally, the supernatants were centrifuged at 221,000 × *g* for 60 min to obtain the vesicle pellets. The pellets were resuspended in a small volume (0.1 to 0.5 ml) of the same buffer and briefly sonicated for use as the membrane vesicles.

### Analytical Methods

#### Dot-Spot Test

The cells were cultivated in a potato medium at 30 or 37°C until the turbidity reached 150–200 Klett units. Then, 1-ml of culture was collected in an Eppendorf tube and centrifuged at 10,000 × *g* for 5 min. The cell pellets from different strains were suspended in 1 ml of 0.85% NaCl solution, and further adjusted by adding NaCl (0.85%) solution to equalize the OD_600_ values. The cell suspensions were then diluted to 10^–1^, 10^–2^, 10^–3^, and 10^–4^ with 0.85% NaCl solution, and 7-μL aliquots were spotted on plates containing three different media, YPG, YPGAE, or YPGDE, and cultured at different temperatures.

#### Measurement of Cell Size

The cells were cultivated in 100 ml of the YPG, YPGAE, or YPGDE medium in a 500-ml Erlenmeyer flask at different temperatures at 200 rpm. The cells were harvested in the early to mid-log phase (a Klett unit of ∼150 and/or acidity of ∼2.%) by centrifugation at 10,000 × *g* for 5 min, washed with 1 ml of 0.85% NaCl, and resuspended in 0.85% NaCl to an OD_600_ of 1. The cells were placed on a glass slide with a cover slip and observed under a microscope (Eclipse E600, Nikon, Tokyo, Japan) at 400 × magnification, with 3--5 images obtained for each sample. The length of approximately 200 cells was measured using PhotoRuler ver. 1.1.3^1^ or ImageJ software (Rasband, W. S., U. S. National Institutes of Health, 1997–2015), and shown as a Box plot, which is composed of a box and a whisker (Dekking et al., 2005). The latter exhibits the upper and lower limits of almost all plots except for some extraordinary plots, which are shown with a circle of each plot, and the box doses the upper and lower limits of averaged plots except for the upper and lower quartile plots (each 25% upper and lower exceptional plots). The median value of the whole plot is depicted as a line inside each box.

#### Measurement of Intracellular Reactive Oxygen Species Levels

The cells were grown at 30°C or 33 in 100 ml of the YPG or YPDAE medium containing 2-μM H_2_DCFDA (dichlorodihydrofluorescein diacetate) as the fluorescence probe in a 500-ml baffled flask. The cells were harvested at the early to mid-log phase by centrifugation at 10,000 × *g* and 4°C for 5 min and washed two times with a 10-mM KPi buffer (pH 7.0). The cells were resuspended in the same buffer at a concentration of 4 ml/g of wet cells and passed two times through a French pressure cell press (American Instrument Co., Silver Spring, MD, United States) at 16,000 psi. After centrifugation at 10,000 × *g* for 10 min to remove intact cells, the supernatant was ultracentrifuged at 100,000 × g for 60 min. The fluorescence intensity of the resulting supernatant was measured at 25°C with excitation at 504 nm and emission at 524 nm. Fluorescence intensity obtained at the same scale or condition was normalized to the protein concentration of the sample used.

#### Lipid Determination

The lipid content of the membrane vesicles was determined with FM 4-64 (Molecular Probes, Eugene, OR, United States), a lipophilic styryl dye, based on the fact that FM 4-46 fluoresces quickly and in a concentration-dependent manner by binding to liposomes (Wu et al., 2009). The vesicle samples were mixed with 5-mM FM 4-64 in a 50-mM HEPES buffer (pH 7.5), and fluorescence was measured with 630-nm emission and 472-nm excitation in a Hitachi 650 10S fluorescence spectrometer. The vesicle lipid concentration was calculated from a calibration curve prepared with 0–25-mg azolectin. The emission and excitation peaks used were determined to be optimal for both vesicle lipid and azolectin.

#### ppGpp Measurement

Cell cultures were rapidly filtrated with PVDF membrane, washed, and then extracted by lysis solvent (methanol:acetonitrile:water = 2:2:1), basically according to the published methods (Varik et al., 2017; Ahmad et al., 2019). And then, ppGpp was analyzed by MonoQ HPLC column chromatography as shown in Supplementary Figure 3.

#### Protein Determination

Protein content was determined using a modified Lowry method (Dulley and Grieve, 1975). Bovine serum albumin was used as the standard protein.

### Preparation for Scanning Electron Microscopy

The cells were collected from the cultures in YPG and YPGAE media at the early to mid-log phase and washed two times with a 50-mM KPi buffer (pH 6.5). The cell pellets were fixed with 2.5% glutaraldehyde in the same buffer for 2–3 h. After washing two times with the buffer, the pellets were dehydrated in a series of (50–100%) ascending ethanol concentrations and then washed two times with 100% *t*-butyl alcohol by incubating for 1 h each at 35–37°C. Finally, the samples were immersed again in100% t-butyl alcohol, frozen, and then dried under reduced pressure at 5°C in a freeze-drying device (JEOL JFD-300). Then, the samples were coated with gold (thickness: 20 nm) using an ion-sputtering device (JEOL JFC-1500) and observed under a JEOL JSM-6100 scanning electron microscope (JEOL Ltd., Tokyo Japan) operating at 15 kV.

### Genome Analysis of Adapted Strains

Genome re-sequencing of the ITO-3 strain was performed using the next-generation sequencing platform Illumina HiSeq 2000 platform at Hokkaido System Science Co., Ltd. (Hokkaido, Japan). Genome mapping was performed to detect mutation points by comparing the draft genome of ITO-3 with the complete genome of the wild-type strain (Ogino et al., 2011).

### Transcriptome Analysis

Wild and ITO-1 strains were grown in 100 ml of the following media in a 500-ml Erlenmeyer flask. Cultivation was performed in the YPG medium at 30°C and in the YPGAE medium at 30°C or 34°C at 200 rpm. When the cell growth reached the early to mid-log phase, the cells were collected, and total RNA was extracted using an RNeasy Minikit (Qiagen, Venlo, Netherlands) according to the manufacturer’s protocol, and then treated with Ribozero (Qiagen) to remove rRNA from mRNA.

The sequencing cDNA libraries were generated using the TruSeq RNA sample prep Kit v. 3, and sequencing was performed as pair-end reads with 100 base runs using the Illumina HiSeq 2500 platform at NODAI Genome Research Center (NGRC), Tokyo University of Agriculture (Tokyo, Japan). This was kindly performed by Dr. Yu Kanesaki of the NGRC.

The adapter sequence and low-quality terminal regions of row reads were trimmed using Trimmomatic v. 0.35 (Bolger et al., 2014). The resulting paired-end reads generated from the high-throughput sequencing were mapped to the complete genome sequence of *K. medellinensis* NBRC 3288 (GenBank accession: AP012166) as the reference with the program Bowtie2 package using the default setting (Ogino et al., 2011; Langmead and Salzberg, 2012). Read counts were calculated using the htseq-count command included in the HTSeq package based on the gene annotation of the NBRC 3288 genome sequence (Anders et al., 2015). The reads per kilobase million mapped fragments (RPKM) were estimated from read counts per gene using an in-house ruby script. The read counts per gene were used as inputs for the following analysis.

Differentially expressed genes (DEGs) were identified using the edgeR package after normalization using the TCC package (Robinson et al., 2010; Sun et al., 2013). The edgeR package uses a false discovery rate (FDR) to determine DEGs. In this study, genes with FDR < 0.05, and log | (fold change)| < 1 were considered DEGs.

## Results

### Thermal Adaptation of *Komagataeibacter medellinensis* Under Acetic Acid Fermentation Conditions at Higher Temperatures

The mesophilic acetic acid bacterium, *K. medellinensis* NBRC 3288, produces high quantities of acetic acid at 30°C, but the production diminishes at 34°C, as the growth and fermentation abilities of the NBRC 3288 strain are hindered at higher temperatures. To obtain a strain that can produce acetic acid at higher temperatures, thermal adaptation was performed under fermentation conditions (see Supplementary Figure 1), where the culture medium contained acetic acid and ethanol, as described in Materials and methods. Thus, three adapted strains were obtained: one at each adaptation step from 34 to 35°C and named ITO-1 to ITO-3. The growth and acetic acid production abilities of these thermally adapted strains were compared at three different temperatures under fermentation conditions (in the YPGAE medium) (Figure 1). As shown, all the adapted strains, but not the wild strain, grew well at 34°C, while only the ITO-3 strain could grow at 35°C. In addition, acetic acid productivity was observed concomitant with their growth behavior. However, turbidity did not always correlate with acetic acid production for reasons described later. In addition, during this thermal adaptation, the tolerance of the adapted strains to ethanol and/or acetic acid, which may be related to the fermentation ability, was also increased (Figure 1B). The fermentation rate of the wild-type strain, in the presence of 1% acetic acid, was considerably reduced with 5% ethanol, but not with 4% ethanol, while all the adapted strains fermented well without any lag time with 5% ethanol in the presence of 1% acetic acid. On the other hand, with 6% ethanol in the presence of 1% acetic acid, only ITO-3 could ferment well without any delay. In addition, the presence of 2% acetic acid also reduced the fermentation rate of the wild-type strain, even with only 3% ethanol, whereas the ITO-3 strain could ferment up to 4% ethanol in the presence of 2% acetic acid without any delay. Regardless, even after adaptation, the maximum acetic acid yield from NBRC 3288 did not exceed 5%, even when a much higher substrate concentration was used that theoretically enabled the generation of acetic acid.

### Comparison of Cell Size and a Cell Surface Between Wild and the Adapted Strains

As shown in Figure 1, cell turbidity and acetic acid productivity were not well correlated; at 30°C, the strains differed in turbidity, despite showing similar acetic acid fermentation ability. In addition, at 35°C, the ITO-3 strain exhibited higher acetic acid productivity, despite exhibiting relatively low turbidity. Therefore, cell sizes were compared between the wild-type and adapted strains (Figures 2A,B). Among wild-type, ITO-1, and ITO-3 strains grown on YPGAE (fermentation conditions) at 30°C, the cell sizes of wild-type and ITO-1 cells were almost identical, but ITO-3 cells were a little shorter than the others. In contrast, when grown at 34°C, cell elongation was observed in the wild-type strain but not in ITO-1 and ITO-3. Contrary to their growth on the YPGAE medium, the wild-type strain grew better than ITO-1 on the YPG medium (non-fermentation conditions), especially at higher growth temperatures (Figure 2C). Therefore, the cell sizes of wild and ITO-1 strains grown on the YPG medium were also compared (see Figure 3D). No cell elongation was observed in either strain at 30°C; in fact, wild-type cells were even shorter than the ITO-1 cells. However, at 34°C, cell elongation was observed to occur in both strains, with ITO-1 cells elongated slightly more than wild-type cells.

We also compared the cell surface morphology of wild-type and ITO-1 strains by Scanning Electron Microscopy (SEM). As shown in Figure 2D, a difference in the cell surface was observed between wild and ITO-1 strains grown in the YPGAE medium at 30°C under fermentation conditions; the wild-type strain exhibited a rough surface with some attached vesicles, while ITO-1 exhibited a smooth surface with fewer vesicle-like structures. At 34°C, vesicle-like structures were increased even in the ITO-1 strain, while many debris or vesicle-like structures were seen together with a few intact cells in the wild-type strain. In contrast, under non-fermented conditions (30°C), both strains exhibited smooth cell surfaces and no vesicle-like structures were found. Thus, stress conditions, such as acetic acid production or high growth temperature, appeared to affect the cell surface structure and generate vesicle-like structures on the cell surface.

Thus, *K. medellinensis* thermally adapted under fermentation conditions appears to acquire robustness at higher temperatures in the presence of acetic acid and/or ethanol through genome modification. In the following studies, we focused on the characterization of ITO-1 strain because it had a limited and simple genetic modification as described below.

### Stress Response Ability of Wild and ITO-1 Strains Under Fermentation Conditions or High Temperatures

**FIGURE 4:**
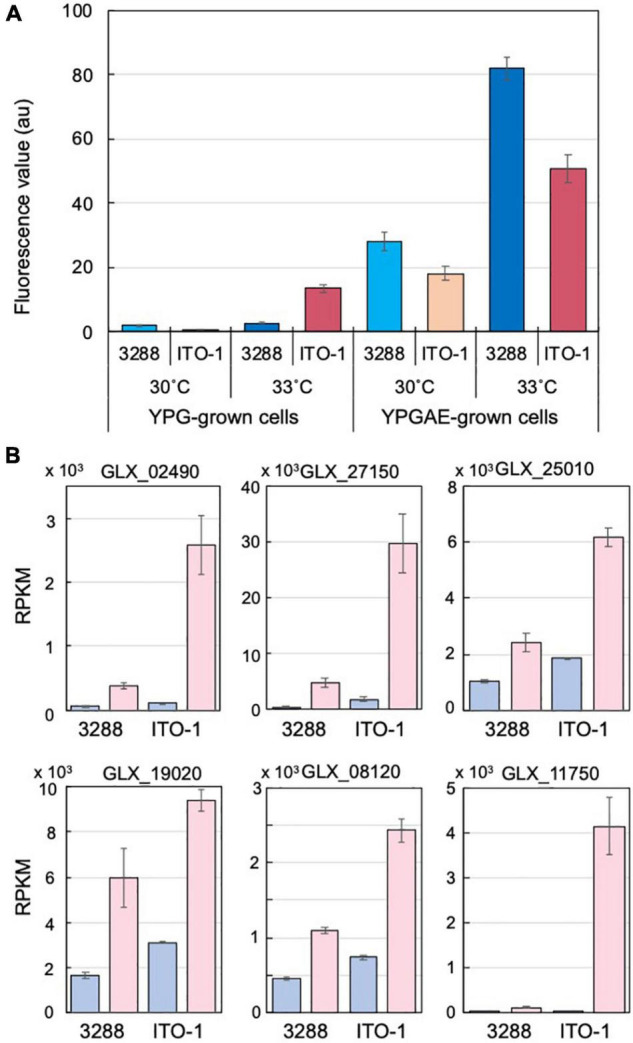
Comparison of stress responses in NBRC3288 and ITO-1 strains grown on YPG and YPGAE media. **(A)** ROS generation was measured, as described in section “Materials and Methods,” in NBRC3288 and ITO-1 strains grown on YPG and YPGAE media at 30°C and at 33°C. The fluorescence values obtained are shown by converting the values to one at the same scale or condition. Error bars represent the standard deviation derived from three independent experiments. **(B)** Typical stress response genes were identified from the transcriptome analysis data of NBRC3288 (wild-type strain) and ITO-1 grown on YPG (blue columns) and YPGAE (pink columns) media at 30°C (Supplementary Table 3), and their RPKM values are shown. GLX_02490, GLX_27150, GLX_25010, GLX_19020, GLX_08120, and GLX_11750 were annotated as peroxidase, Hsp20, RpoH, DegP, G6P dehydrogenase, and NADPH-dependent aldehyde dehydrogenase, respectively. Error bars represent the standard deviation of RPKM values derived from three different samples.

Cells adapted to high temperatures are expected to show a stress-tolerant phenotype, and cells grown under fermentation conditions may also have stress tolerance ability to cope with the substrate ethanol and product acetic acid, both of which are toxic to microbial cells. Such stress tolerance may be related to the generation of reactive oxygen species (ROS). Microbial cells generate a large amount of ROS, especially when grown at growth-limiting high temperatures (Davidson et al., 1996; Matsushita et al., 2016; Chang et al., 2017; Nantapong et al., 2019), and thermally adapted strains have been shown to generate less ROS than the wild-type strain when grown at higher growth temperatures (Matsumoto et al., 2018, 2020). Acetic acid fermentation may similarly generate ROS due to structural damage to the cell membrane caused by acetic acid and/or ethanol. In the present study, the cells grown under fermentation conditions (YPGAE medium) showed extremely higher ROS levels than those grown under non-fermentation conditions (YPG medium) (Figure 4A). ROS generation was also increased in the YPGAE medium at higher temperatures in both strains. In addition, ROS generation was repressed in the ITO-1 strain compared to the wild-type strain in the YPGAE medium, but not in the YPG medium at 33°C (Figure 4A). Thus, to determine the mechanism behind ROS reduction under fermentation conditions and at higher growth temperatures, transcriptome analysis was performed with the NBRC 3288 strain and the adapted ITO-1 strain. RNA expression levels of both strains were compared during growth on the YPG medium and the YPGAE medium at 30°C, and also of ITO-1 between the cells grown at 30°C and 34°C on the YPGAE medium. Under fermentation conditions, a large number of stress response genes, such as ROS scavenging enzymes, heat shock proteins, chaperonins, and NADPH-generating enzymes, were upregulated in both strains (Supplementary Table 3). Typically, several stress response genes upregulated in the fermentation conditions, including peroxidase, Hsp20, DegP, RpoH, G6P dehydrogenase, and aldehyde dehydrogenase, showed a more substantial increase in ITO-1 than in the wild-type strain (Figure 4B). On the other hand, when the temperature increased to 34°C, enzymes related to the energy generation system, such as *aptABCDEFGH* (F_1_F_0_-ATP synthase), *nuoDEFGHIJKLN* (Type I NADH dehydrogenase), *cyaABCD* (H^+^-pumping ubiquinol oxidase), *adhAB* (PQQ-alcohol dehydrogenase), and *aldFGH* (membrane-bound aldehyde dehydrogenase), which were repressed under fermentation conditions, were expressed at higher levels in the ITO-1 strain (Supplementary Table 3). Thus, repression of radical generation due to fermentation and energy generation at higher temperatures could be important to survive such stress conditions.

### Genome Analysis of Adapted Strains

To determine how thermal adaptation affects the genome, the ITO-3 draft genome was sequenced and compared with the complete genome of the NBRC3288 strain to detect mutations (Ogino et al., 2011). A total of eight mutations, including three deletions, one insertion, and four nucleotide substitutions, were detected in ITO-3 (Table 1). Using PCR sequencing, all eight mutations were confirmed in ITO-3, and it was shown that wild, ITO-1 and ITO-2 strains have zero, three, and six mutations of the eight mutations found in ITO-3, respectively. Thus, three mutations occurred in ITO-1, with three and two additional mutations occurring in ITO-2 and ITO-3 strains, respectively (Table 1). Some of the mutations seem to confer thermotolerance to the wild strain, while some may have occurred spontaneously by chance. In this study, we focused on mutations in the ITO-1 strain, which has a growth advantage, compared with the wild strain, on the YPGAE medium at 34°C (Figure 1), and thus has the ability to produce acetic acid, coping with heat stress as well as ethanol and/or acetic acid stress. ITO-1 had mutations in three genes, with a 12-bp deletion, 12-bp insertion, and a 424-bp deletion in GLX_00040 (*gyrB*), GLX_19020 (*degP*), and GLX_25720 (*spoT*), respectively (Table 1). These genes correspond to the enzymes DNA gyrase, periplasmic endopeptidase, and GTP pyrophosphokinase, respectively (Table 1). Among these genes, the *gyrB* mutation was not investigated in this study, because its overexpression had no effect on the growth of the wild and ITO-1 strains (Supplementary Figure 2), and disruption of the gene may not be possible due to its indispensability. Thus, in this study, we focused on two genes, *degP* and *spoT*.

**TABLE 1:** Mutated genes and the mode of mutations in the adapted strains, ITO-1, ITO-2, and ITO-3.

Orf ID	Product	Mutation				ITO-1	ITO-2	ITO-3
		Position	Original sequence	Mutated sequence	Mutational Pattern			
GLX_00040	DNA gyrase subunit B (*gyrB*)	19286	CGCCGCGCGTGAG	C	12 bp deletion	🌑	🌑	🌑
GLX_00820	Hypothetical protein	96323	G	A	D239N			🌑
GLX_07330	Hypothetical protein	843150	T	TG	1 bp insertion		🌑	🌑
GLX_19020	Endopeptidase DegP/Do (*degP*)	2118313	G	GCTTTCCCTTCCC	12 bp insertion	🌑	🌑	🌑
GLX_21880	α, α-trehalose-phosphate synthase	2417396	C	T	A342V		🌑	🌑
GLX_25720	GTP pyro-phosphokinase (*spoT*)	2850572–2850989			424 bp deletion	🌑	🌑	🌑
GLX_25830	Outer membrane protein	2862183	C	T	R812C			🌑
Intergenic	Upstream of GLX_26500	3136818	G	A	SNP		🌑	🌑

*🌑, Mutations occurred in this mutant; 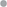, Mutations retained in these mutants.*

### Effect of *degP* Mutation on Thermotolerance of *Komagataeibacter medellinensis* NBRC 3288

ITO-1 has a mutation in the *degP* gene, which generates a mutated DegP with additional 4 amino acid residues in the protein sequence. The mutated DegP may play an important role in the cell survival under high temperature or fermentation conditions. As DegP is known to function as a cell surface chaperone or protease (Spiess et al., 1999) and defects in DegP lead to the generation of membrane vesicles (Schwechheimer et al., 2013), the DegP mutation may be involved in modulating the cell surface structure. In fact, such cell surface changes were observed in the wild-type strain but reduced in the ITO-1 strain under stress conditions (Figure 2D). This phenomenon could be related to the DegP mutation, which may influence the growth of the ITO-1 strain on the YPGAE medium.

**FIGURE 5:**
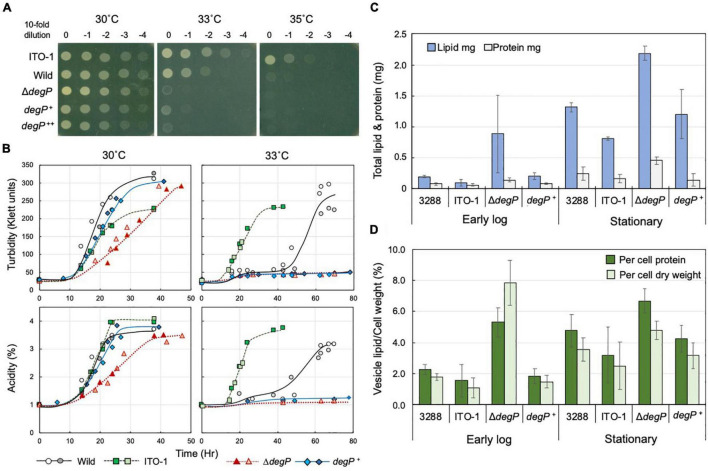
Comparison of cell growth and membrane vesicle generation in NBRC3288, ITO-1, and *degP*-mutated strains grown under fermentation conditions. **(A)** NBRC 3288 (wild-type), ITO-1, and Δ*degP* strains harboring plasmid pMV24, and Δ*degP* strains harboring pP*degP*^WT^ and pP*degP*^ITO–1^ (shown as *degP*^+^ and *degP*^++^, respectively) were cultivated in a potato medium containing 10-mg/ml ampicillin (except for wild and ITO-1 strains) at 30°C. The dot-spot test was then performed, as described in Materials and methods, on YPGAE agar plates at different temperatures. **(B)** NBRC 3288 (wild-type), ITO-1, Δ*degP* strains, and Δ*degP* strains harboring plasmids pMV24, pP*degP*^WT^ (*degP*^+^), or pP*degP*^ITO–1^ were cultivated in 100-ml of the YPGAE medium in a 500-ml-baffled flask at 30 and 33°C. Typical results are shown here from more than three times independent cultures of each strain. As cultures of Δ*degP* (pMV24) and ΔdegP (pP*degP*^ITO–1^) at 30°C grew almost identical to Δ*degP*, *degP*^+^, and also Δ*degP* (pP*degP*^ITO–1^) at 33°C to *degP*^+^ at 33°C, their growth data are not shown here. **(C)** NBRC3288 (wild-type), Δ*degP*, and Δ*degP* harboring pP*degP*^WT^ (*degP*^+^) were cultivated in 100-ml of the YPGAE medium in a baffled flask at 30°C till the early log phase (2.1–2.5% acidity) or till the late log to the stationary phase (3.5–4% acidity). Membrane vesicles were prepared from culture supernatants, as described in section “Materials and Methods.” Then, lipids and proteins were determined as described in section “Materials and Methods” and shown as the total content of the whole culture supernatant. Error bars represent the standard deviation derived from three independent experiments. **(D)** Total lipid content is shown as the value (%) per cell protein content or dry cell weight. Lipid content (mg) was the same as shown in **(C)**, and total cell protein (mg) or dry cell weight (mg) was determined from the cell pellets after removal of the culture supernatants, which were used for the preparation of the membrane vesicles. Error bars represent the standard deviation derived from three independent experiments.

To determine the role of DegP in cell growth under fermentation conditions, its disruptantΔ*degP* was compared with the wild-type and ITO-1 strains in dot-spot analysis (see Figure 3B). The Δ*degP* strain grew at 30°C as well as other strains, but not at 34°C on the YPGAE medium. We further studied the effect of *degP* overexpression with a plasmid, pP*degP*^WT^ or pP*degP*^ITO–1^, containing wild-type or mutated *degP* genes, respectively. pP*degP*^WT^, but not pP*degP*^ITO–1^, exhibited a marginal effect on the recovery of the disturbed cell growth of Δ*degP* at 33°C (Figure 5A). These plasmids probably produced too much DegP enzymes, especially mutated one, because the cell growth was largely disturbed when expressing them in wild-type and ITO-1 strains (Supplementary Figure 2).

When Δ*degP*, wild-type, and ITO-1 strains were grown in Erlenmeyer flasks on the YPGAE medium, there was no clear growth difference at 30°C, whereas at 33°C, only ITO-1 grew well, and the wild and Δ*degP* strains exhibited delayed growth and no growth, respectively (data not shown). The strains were then cultured in baffled flasks, which facilitate high aeration conditions to stimulate cell growth. Here, the Δ*degP* strain exhibited some growth defects at 30°C compared with wild-type and ITO-1 strains, and the Δ*degP* strain harboring pP*degP*^WT^, but not pP*degP*^ITO–1^, grew better than Δ*degP* (Figure 5B). In contrast, only ITO-1 grew well at 33°C, whereas the wild strain exhibited delayed growth, and both the Δ*degP* strain and the strains harboring pP*degP*^WT^ or pP*degP*^ITO–1^ did not grow (Figure 5B).

DegP may be related to the cell surface structure as shown above and has been shown to be involved in membrane vesicle generation (McBroom et al., 2006; McBroom and Kuehn, 2007). Therefore, to determine the role of DegP in cell growth, all the above strains were grown with high aeration (baffled flask) at 30°C under fermentation conditions, and membrane vesicle generation was compared. Membrane vesicles were isolated from the culture supernatants, as described in section “Materials and Methods,” and their lipid and protein contents were examined; they exhibited a fairly high lipid/protein ratio of 1.6∼8.4 compared to the cell membranes (lipid/protein ratio of ∼0.6 to 0.8). As shown in Figure 5C, the membrane vesicle content increased from the early log phase (2.1–2.5% acidity) to the stationary phase (3.5–4% acidity) in all the strains. In particular, the Δ*degP* strain produced considerably more vesicles than the wild-type strain. When compared with the rate of vesicle generation per biomass (Figure 5D), Δ*degP* strain actually produced more, whereas the ITO-1 strain repressed vesicle generation compared to the wild-type strain at all growth phases. Furthermore, the inclusion of pP*degP*^WT^ also reduced the vesicle production observed in the Δ*degP* strain. Thus, defects in DegP may induce vesicle generation under stress conditions, such as acetic acid production, and *degP* mutation in ITO-1 may have some gain of function to reduce vesicle generation.

### Effect of *spoT* Mutation in the Thermotolerance of ITO-1 Strain

ITO-1 has a mutation in the *spoT* gene, which may generate a C-terminally truncated SpoT protein (Figure 3A). Therefore, we first prepared a deletion mutant of the whole SpoTC-terminal regulatory domain (CTD) by inserting the Tc^R^ cassette between the N-terminal catalytic domain and the CTD (Figure 3A). Then, the growth of the resultant disruptant, ΔTGSAct, was compared with that of wild-type and ITO-1 strains on the YPG and YPGAE media at 30, 34, and 35°C by dot spot (Figure 3B). Although the growth of the ΔTGSAct strain was slightly worse than that of the other strains at 30°C, it grew well at high temperatures on both media. In contrast, the wild-type strain grew better on the YPG medium and the ITO-1 strain on the YPGAE medium at higher temperatures. Thus, the growth of the ΔTGSAct strain was similar to that of well-growing strains on each medium: wild-type strain on YPG and ITO-1 strain on YPGAE. In terms of cell length, the wild-type strain elongated at a higher growth temperature (34°C) on both media, but cell elongation was observed in the ITO-1 strain only on the YPG medium (Figure 3D). Therefore, the growth in flask culture and cell length of the ΔTGSAct strain were compared with those of the wild-type and ITO-1 strains on YPG and YPGAE media (Figures 3C,D). On YPG medium, the ΔTGSAct strain exhibited lower turbidity than the other strains at 30°C, but the turbidity was retained at 34°C, unlike the wild-type and ITO-1 strains, both of which displayed reduced growth and elongated cells at 34°C. On the YPGAE medium, the ΔTGSAct strain grew well both at 30 and 34°C, similar to the ITO-1 strain, whereas the wild-type strain showed considerably delayed growth at 34°C. Concomitant with the cell growth behavior, the ΔTGSAct strain maintained a shorter cell length and did not elongate at all, like ITO-1, on YPGAE at 34°C. These results suggest that the truncated SpoT in ΔTGSAct, similar to the mutated SpoT in ITO-1, may repress cell elongation or reduce cell size as observed in both ITO-1 and ΔTGSAct strains. This phenomenon may be mediated by (p) ppGpp accumulation, because it has been shown that the CTD of RelA represses ppGpp synthesis (Gropp et al., 2001; Mechold et al., 2002; Irving and Corrigan, 2018), and that cell size reduction is induced by ppGpp (Schreiber et al., 1995; Stott et al., 2015; Vadia et al., 2017).

Therefore, we examined the ppGpp content of these strains grown on the YPG and YPGAE at 30°C. The nucleotides were extracted directly from the intact cells and analyzed by anion-exchange (Mono-Q column) HPLC, where ppGpp and other nucleotides could be well separated (Supplementary Figure 3). However, due to the very low content of ppGpp in the cells compared with other nucleotides, relatively large amounts of culture (80–250 ml) were needed for the extraction before application to HPLC. This is probably due to the difficulty of cell lysis in *K. medellinensis* cells, especially those grown on the YPGAE medium, because *Gluconacetobacter europaeus* (now *Komagataeibacter europaeus*) (Trcek et al., 2007) and *A. pasteurianus* (Kanchanarach et al., 2010) cells grown on high acetic acid concentrations have been shown to be covered with spongy and amorphous materials, respectively, and become tight and hard. Thus, although a quantitative comparison was very difficult among the strains, especially because no data were obtained from the wild cells grown on the YPGAE medium, the ΔTGSAct strain grown on YPG or ITO-1 strain grown on YPGAE exhibited increased levels of ppGpp when compared to other nucleotides like GDP or ATP extracted from the same cells. Thus, it may be said that phenotypic changes, such as cell size reduction observed in the ITO-1 strain and the ΔTGSAct strain, could be induced by increased alarmone production on account of the truncated SpoT protein.

However, there was a difference in growth between ΔTGSAct and ITO-1, especially under non-fermentation conditions (YPG medium) (Figures 3B,C). This is probably because only the Act-4 domain in the CTD is deficient in ITO-1, while both TGS and Act-4 domains are deficient in ΔTGSAct (Figure 3A). Therefore, we also examined the effect of the Act-4 domain on growth or cell phenotypes.

### Deletion of the Act-4 Domain in *Komagataeibacter medellinensis* NBRC 3288

**FIGURE 6:**
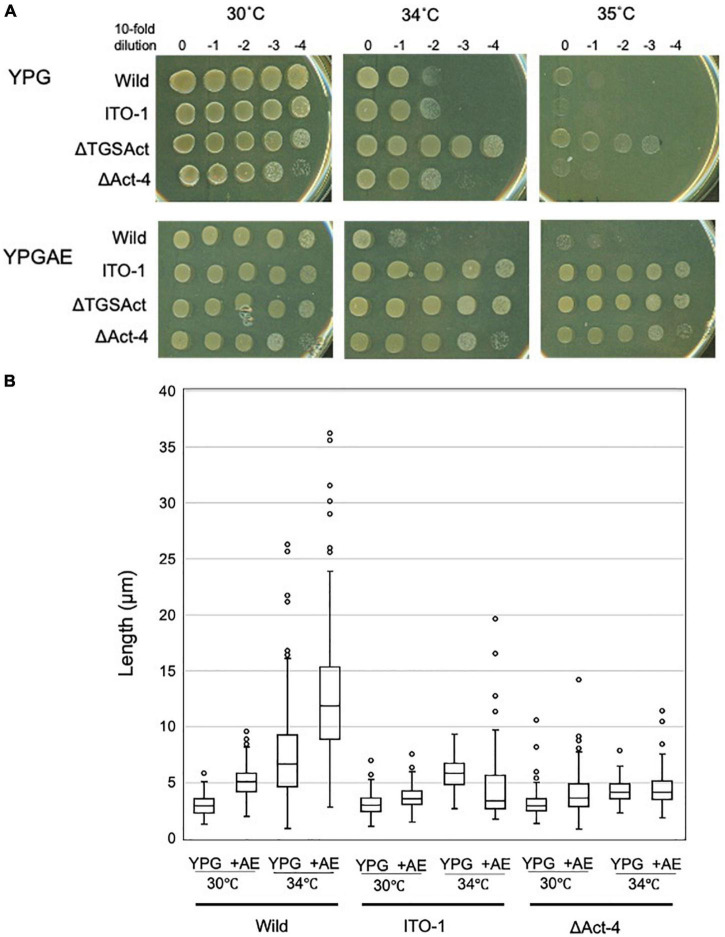
Cell growth and cell length of SpoT mutant strain, ΔAct-4. **(A)** NBRC 3288 (wild-type), ITO-1, ΔTGSAct, and ΔAct-4 strains were cultivated in the potato medium containing appropriate antibiotics (25-mg/ml tetracycline for ΔTGSAct and ΔAct-4 strains), and dot-spotted, as described in Materials and methods, on YPG or YPGAE agar plates, and then incubated at 30, 34, and 35°C. **(B)** Cell length was measured in wild-type, ITO-1, and ΔAct-4 strains grown on YPG or YPGAE (+AE) at 30 and 34°C. Box plots are shown by using the PhotoRuler, as described in Materials and methods, and the median values of each cell grown in YPG were 3 (30°C) and 6.6 μm (34°C) in wild-type, 3.1 (30°C) and 5.9 μm (34°C) in ITO-1, and 3 (30°C) and 4.2 μm (34°C) in ΔAct-4, and those of each cell grown in YPGAE were 5.1 (30°C) and 11.9 μm (34°C) in wild-type, 3.6 (30°C) and 3.5 μm (34°C) in ITO-1, and 3.8 (30°C) and 4.3 μm (34°C) in ΔAct-4.

To determine the functional difference between the Act-4 domain and the whole regulatory domain (CTD), the ΔAct-4 mutant was prepared by inserting the Tc^R^ cassette between the TGS domain and Act-4 domains (Figure 3A). Then, the growth of ΔAct-4 strain was compared with that of the ΔTGSAct, wild-type, and ITO-1 strains by dot spot on both non-fermentation (YPG) and fermentation (YPGAE) media at 30, 34, and 35°C (Figure 6A). On the YPGAE medium, the ΔAct-4 strain grew better than the wild-type strain at 34 and 35°C but was slightly worse than the ΔTGSAct and ITO-1 strains. In contrast, on the YPG medium, the ΔAct-4 strain grew worse than the ΔTGSAct strain at 34 and 35°C but similar to the wild-type and ITO-1 strains. Thus, the ΔAct-4 strain exhibited thermotolerance under fermentation conditions although slightly weaker than the ITO-1 and ΔTGSAct strains, but not under non-fermentation conditions, like ITO-1 but unlike ΔTGSAct. However, when the cell length was compared, the ΔAct-4 strain displayed a shorter cell length than the wild and ITO-1 strains but almost the same cell length as ΔTGSAct (Figures 3D, 6B) under non-fermentation conditions at 34°C, although thermotolerance was not observed unlike the ΔTGSAct strain.

### Effect of Act-4 Domain Deletion in *SpoT* in *Acetobacter pasteurianus* SKU1108

**FIGURE 7:**
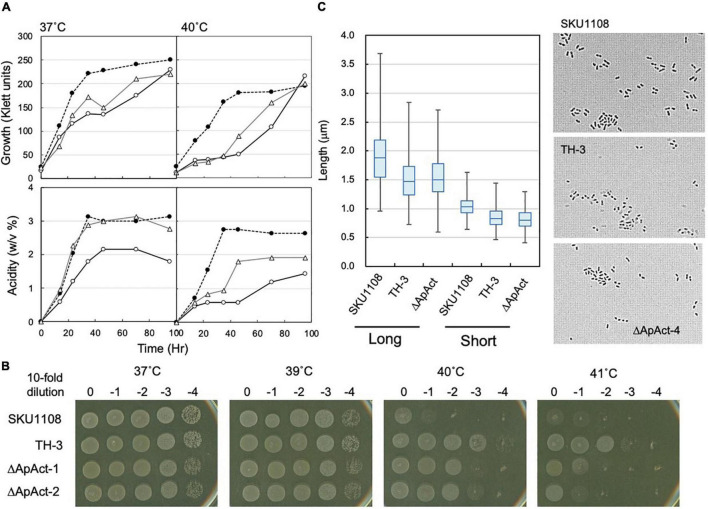
Effect of *spoT* mutation (ΔApAct) on the growth and cell length in *Acetobacter pasteurianus* SKU1108. **(A)** SKU1108 (wild-type), TH-3 (thermally adapted strain), and ΔApAct (Act-4-deleted mutant) were cultivated in 100 ml of the YPGDE medium (containing 4% ethanol) in a 500-ml Erlenmeyer flask at 37 and 40°C. Pre-cultures of these strains were grown in 5 ml of the potato medium at 37°C. Typical results are shown here from three independent cultures of each strain. **(B)** Comparison of dot-spot growth among *A. pasteurianus* SKU1108, TH-3, the same two *spoT* mutant ΔApAct strains, ΔApAct-1 and ΔApAct-2, on the YPGDE agar medium. *A. pasteurianus* strains were cultivated in the 5 ml potato medium at 37°C until 100 Klett units. The strains were diluted with the YPGD medium, spotted on YPGDE (4% ethanol) agar plates, and then incubated for 48 h at 37, 39, 40, and 41°C. **(C)** Cell length was measured in SKU1108, TH-3, and ΔApAct strains grown on the YPGDE medium at 37°C till the mid-log phase (Klett units and acidity of SKU1108, TH-3, and ΔApAct were 135 and 1.2%, 130 and 1.2%, and 100 and 1.2%, respectively). Both the long and short axes of the cells are shown as the box plot by using ImageJ software, as described in section “Materials and Methods.” The median values (long axis) of each cell were 1.87, 1.47, and 1.48 μm in SKU1108, TH-3, and ΔApAct, respectively.

To confirm the role of the Act-4 domain, a similar deletion mutant strain (ΔApAct) was created in another acetic acid bacterium, *A. pasteurianus* SKU1108, *via* a markerless mutation. *A. pasteurianus* SKU1108 is a naturally thermotolerant strain that can perform acetic acid fermentation at 37°C. This strain was further adapted to higher temperatures till 40°C under acetic acid fermentation conditions (the YPGDE medium; the YPGD medium containing 4% ethanol) to generate a thermally adapted strain, TH-3 (Matsutani et al., 2013). As shown in Figure 7A, the adapted TH-3 strain showed higher growth and fermentation ability even when grown on the YPGDE medium at 37°C, although a more critical difference was observed at 40°C. The TH-3 strain has 11 mutations, of which the transcriptional regulator MarR, amino acid transporter, C_4_-dicarboxylate transporter, and PII uridylyltransferase were shown to be involved in fermentation ability and thermotolerance (Matsutani et al., 2013, unpublished), but it did not have a *spoT* gene mutation. The *spoT* genes of SKU1108 and *K. medellinensis* are homologous to each other, and a sole gene functions as an RSH-enzyme in these a-Proteobacterial strains (data not shown). Therefore, SpoT in SKU1108 was expected to have a function similar to that in *K. medellinensis*. As shown in Figures 7A,B, the ΔApAct strain also showed higher thermotolerance under fermentation conditions. In addition, the ΔApAct strain similar to the TH-3 strain showed a shorter cell length than that of the wild-type SKU1108 strain (Figure 7C). Thus, the acetic acid bacterium *A. pasteurianus*, even though from a different genus than *K. medellinensis*, could also acquire thermotolerance *via* the deletion of one of the regulatory domains Act-4 of SpoT protein, which would induce a shorter cell length.

## Discussion

In this study, high-temperature-adapted strains, ITO-1, ITO-2, and ITO-3, were obtained sequentially from *K. medellinensis* NBRC3288 by an experimental evolution approach in acetic acid fermentation conditions, where the cells grew at increased temperatures under high concentrations of ethanol and acetic acid. The adapted strain ITO-1 analyzed in this study showed robustness, exhibiting thermotolerance as well as tolerance to resist acetic acid and/or ethanol, while simultaneously exhibiting a trade-off of reduced growth than the wild-type strain under non-fermentation conditions (in the absence of ethanol and/or acetic acid). The adapted ITO-1 strain showed two typical morphological phenotypes related to cell surface structure and cell length. The wild-type NBRC3288 exhibited a rough or irregular cell surface structure and marked cell elongation at higher temperatures, especially under acetic acid fermentation conditions. In contrast, ITO-1 acquired the ability to exhibit a smoother cell surface and smaller cell length than the wild-type strain, even at 30°C. Related to the phenotypic changes, in this study, we focused on the mutations of two genes, *degP* and *spoT*, acquired during the experimental evolution of the ITO-1 strain.

### The Role of *degP* Mutation in ITO-1 Strain

Scanning electron microscopy observation indicated that the wild strain grown under fermentation conditions had an irregular cell surface, with attached membrane vesicle-like structures, whereas ITO-1, having a *degP* mutation, exhibited a relatively smooth cell surface where the production of such vesicles may be suppressed. Thus, the membrane vesicle generation was examined under the fermentation conditions employed in this study. The Δ*degP* strain produced a larger number of vesicles than the wild-type strain, whereas the ITO-1 strain repressed vesicle generation throughout its growth. Thus, as expected, a defect in DegP could induce vesicle generation under stressful fermentation conditions, while the mutated DegP in ITO-1 may gain a higher enzyme activity, albeit no direct evidence, to enable the cells to stabilize against such envelope stress by deleting some denatured or abnormal proteins generated on the cell surface. The stable cell surface, including the outer membrane and the periplasm, may confer the cells the ability to tolerate several stresses, especially at higher growth temperatures.

### The Role of *spoT* Gene Mutation in ITO-1 Strain

SpoT has four domains: degradation and synthesis domains in the N-terminal half (the catalytic domain) and TGS and Act-4 domains in the C-terminal half (regulatory or the CTD domain) (Figure 3A). The ITO-1 strain has a truncated SpoT, which lost a part of the CTD, including the Act-4 domain, and shows thermotolerance under acetic acid fermentation, but not non-fermentation, conditions at 34°C. The ΔTGSAct mutant, with the SpoT lacking the whole CTD, exhibited thermotolerance under both fermentation and non-fermentation conditions, while the other mutant ΔAct-4, lacking only the Act-4 domain in the *spoT* gene, achieved thermotolerance only under fermentation conditions, of which the phenotype is similar to that of the ITO-1 strain. In correlation with the thermotolerance, cell elongation was repressed in these adapted and mutated strains, as opposed to the cell elongation observed in the wild-type strain irrespective of the culture conditions. The ITO-1 strain could repress cell elongation only under fermentation conditions, but both the ΔTGSAct and ΔAct-4 strains repressed cell elongation under both fermentation and non-fermentation conditions. Thus, at least in both ITO-1 and ΔTGSAct strains, it is reasonable to conclude that the thermotolerance phenotype is linked to the ability to repress cell elongation induced by heat stress and partly by fermentation stress. However, there was some uncertainty with the ΔAct-4 strain, as it could not grow well but repressed cell elongation under non-fermentation conditions at 34°C. Thus, to clarify the uncertainty in the function of the Act-4 domain, a similar “Act-4 domain”-deleted mutant (ΔApAct) was created in another acetic acid bacterium, *A. pasteurianus* SKU1108. Results from the ΔApAct strain clearly showed that the mutated SpoT could confer the strain thermotolerance together with the reduced cell size under fermentation conditions (Figure 7), thus providing additional supporting information that SpoT missing Act-4 domain functions could reduce the cell size, at least under acetic acid fermentation conditions.

**FIGURE 8:**
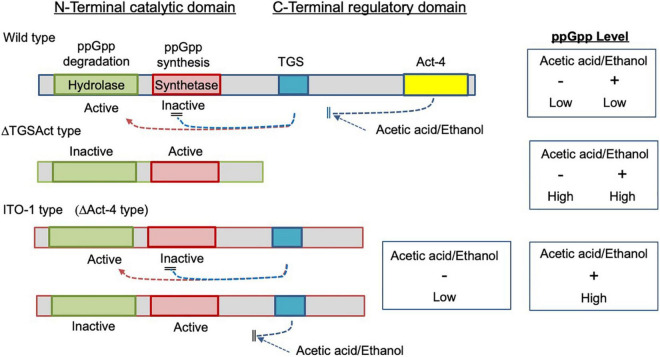
Image for the functional states of the mutated SpoTs from ITO-1 and ΔTGSAct strains. In the wild strain, the hydrolase and synthetase of the catalytic domain (N-terminal) are regulated by the TGS and also by the Act-4 domains (CTD) to be active and inactive, respectively, and thus ppGpp may not be accumulated. In the ΔTGSAct strain, both hydrolase and synthetase of the catalytic domain are derepressed due to the depletion of CTD to be inactive and active, respectively, and thus ppGpp may be accumulated. In the ITO-1 strain, due to the missing of the Act-4 domain, which may control the effect of acetic acid or ethanol, the catalytic domain is regulated solely by the TGS domain, which may be affected by acetic acid or ethanol negatively. Thus, the hydrolase and synthetase of the catalytic domain could be derepressed only in the presence of acetic acid or ethanol.

Thus, it is conceivable that the thermotolerance ability is related to cell size regulation, which may be mediated by (p) ppGpp accumulation because the reduction in cell size has been shown to be induced by ppGpp (Schreiber et al., 1995; Stott et al., 2015; Vadia et al., 2017). In this study, our preliminary data suggested that ITO-1 and ΔTGSAct may accumulate ppGpp to a certain level, at least under fermentation conditions (Supplementary Figure 3). From the phenotypic differences shown above, it is expected that SpoT of ΔTGSAct, missing the whole CTD, may synthesize ppGpp under both growth conditions, while the variant missing only the Act-4 domain, ITO-1, could accumulate ppGpp by derepressing the catalytic domain only in the presence of acetic acid (or ethanol) (Figure 8). Thus, it is conceivable that the ITO-1 strain may acquire the ability to grow better at higher temperatures under fermentation conditions *via* ppGpp-dependent stringent response. This notion is supported by the cell size reduction (Figures 2B, 3D, 6B) and the reduction in expression of translational machinery components, such as ribosomal proteins and t-RNA synthetases under fermentation conditions at 30°C, especially in the ITO-1 strain (Supplementary Table 3). The latter phenomenon has already been shown to occur in ppGpp-dependent stringent responses (Paul et al., 2004; Traxier et al., 2008).

### Adaptation of ITO-1 Strain via *degP* and *spoT* Mutations

In this study, at least two mutations in the *degP* and *spoT* genes were shown to contribute to stable acetic acid fermentation at higher growth temperatures. The *degP* and *spoT* mutations may contribute to the tolerance ability of the ITO-1 strain *via* cell surface stability and stringent response, respectively, as described above. Since disturbances in the biogenesis of cell surface components, such as outer membrane, lipopolysaccharide, or peptidoglycan, are known to induce stringent response (Roghanian et al., 2019), it is reasonable to assume that the robustness acquired by DegP on account of its mutation could work to repair the cell surface structure in combination with the stringent response induced by *spoT* mutation. In the ITO-1 strain, a large number of stress response genes, such as ROS scavenging enzymes, heat shock proteins, chaperonins, and NADPH-generating enzymes, are upregulated under fermentation conditions, and energy-generating enzymes are upregulated at higher growth temperatures. The former expressional change may also be related to *spoT* mutation in ITO-1 because it has been shown that RpoS-dependent stress response genes could be induced by ppGpp both in *E. coli* (Traxier et al., 2008) and the a-Proteobacterium *Rhizobium etli* (Vercruysse et al., 2011), where heat stress genes such as HPS20 and oxidative stress genes are upregulated. These changes may also contribute to stable cell growth or fermentation by reducing ROS generation and increasing energy generation. Thus, the thermally adapted strain, ITO-1, seems to have acquired thermotolerance and acid or alcohol tolerance abilities *via* acquired gene mutations during the experimental evolutionary process.

In conclusion, ITO-1 strain acquired robustness during experimental evolution *via* at least two genes, *degP* and *spoT*, mutations, both of which might be gain-of-function. The former mutation seems to release envelope stress, stabilizing cell surface structure, while the latter seems to induce stringent response, reducing cell size and increasing ROS scavenging ability. The combination of both the phenotypic changes enables ITO-1 to be robust against several fermentation stresses.

## Data Availability Statement

The datasets presented in this study can be found in DDBJ Sequence Read Archive repository, accession numbers, DRA012974, for genome resequence and DRA012979 for transcriptome analysis.

## Author Contributions

KM planned the project. NK, TY, and KM designed the experiments. MO, YM, KI, and NM carried out the experiments with support from NK (genetic work), MM (genome analysis and transcriptome analysis), YK (transcriptome analysis), and ST (microscopy analysis). NK, MM, and NM prepared the manuscript with help from KM and TY. All authors contributed to the scientific discussion throughout the work and have read and approved the manuscript.

## Conflict of Interest

The authors declare that the research was conducted in the absence of any commercial or financial relationships that could be construed as a potential conflict of interest.

## Publisher’s Note

All claims expressed in this article are solely those of the authors and do not necessarily represent those of their affiliated organizations, or those of the publisher, the editors and the reviewers. Any product that may be evaluated in this article, or claim that may be made by its manufacturer, is not guaranteed or endorsed by the publisher.

## Footnotes

1
http://www.inocybe.info/_userdata/ruler/PhotoRuler.html

## Abbreviations

Abbreviations**:** DegP: periplasmic endopeptidase (serine proteinase)
SpoT: GTP pyrophosphokinase
ppGpp: guanosine 3 ′ -diphosphate 5 ′ -diphosphate
RSH: RelA/SpoT homology family
CTD: C-terminal regulatory domain of *spoT* gene
TGS: regulatory domain of SpoT suggested to be ligand binding
Act-4: regulatory domain of SpoT suggested to bind to amino acids and regulate associated enzymes
ROS: reactive oxygen species
RpoH: RNA polymerase sigma factor
RpoB: RNA polymerase β-subunit
Δ TGSAct: a mutant strain of NBRC 3288 lacking the whole CTD in *spoT* gene
Δ Act-4: a mutant strain of NBRC 3288 lacking only the Act-4 domain in the *spoT* gene
Δ ApAct: a mutant strain of SKU 1108 lacking only the Act-4 domain in the *spoT* gene
YPG: culture medium containing 5-g yeast extract 5-g polypeptone and 5-g glycerol per 1 L water
YPGAE: culture medium containing 1% acetic acid and 3% ethanol in the YPG medium.

## References

[B1] Ahmad S. Wang B. Walker M. D. Tran H.-K. R. Stogios P. J. Savchenko A. (2019). An interbacterial toxin inhibits target cell growth by synthesizing (p)ppApp. *Nature* 575 674–678. 10.1038/s41586-019-1735-9 31695193 PMC6883173

[B2] Anders S. Pyl P. T. Huber W. (2015). HTSeq–a Python framework to work with high-throughput sequencing data. *Bioinformatics* 31 166–169. 10.1093/bioinformatics/btu638 25260700 PMC4287950

[B3] Azuma Y. Hosoyama A. Matsutani M. Furuya N. Horikawa H. Harada T. (2009). Whole-genome analyses reveal genetic instability of *Acetobacter pasteurianus*. *Nucleic Acids Res.* 37 5768–5783. 10.1093/nar/gkp612 19638423 PMC2761278

[B4] Bolger A. M. Lohse M. Usadel B. (2014). Trimmomatic: a flexible trimmer for Illumina sequence data. *Bioinformatics* 30 2114–2120. 10.1093/bioinformatics/btu170 24695404 PMC4103590

[B5] Caspeta L. Chen Y. Ghiaci P. Feizi A. Buskov S. Hallström B. M. (2014). Biofuels Altered sterol composition renders yeast thermotolerant. *Science* 346 75–78. 10.1126/science.1258137 25278608

[B6] Chang R. Lv B. Li B. (2017). Quantitative proteomics analysis by iTRAQ revealed underlying changes in thermotolerance of *Arthrospira platensis*. *J. Proteomics* 165 119–131. 10.1016/j.jprot.2017.06.015 28645570

[B7] Charng Y. Liu H. Liu N. Chi W. Wang C. Chang S. (2007). A heat-inducible transcription factor, HsfA2, is required for extension of acquired thermotolerance in *Arabidopsis*. *Plant Physiol*. 143 251–262. 10.1104/pp.106.091322 17085506 PMC1761974

[B8] Charoensuk K. Sakurada T. Tokiyama A. Murata M. Kosaka T. Thanonkeo P. (2017). Thermotolerant genes essential for survival at a critical high temperature in thermotolerant ethanologenic *Zymomonasmobilis*TISTR 548. *Biotechnol. Biofuels* 10:204. 10.1186/s13068-017-0891-0 28855965 PMC5571576

[B9] Chinnawirotpisan P. Theeragool G. Limtong S. Toyama H. Adachi O. Matsushita K. (2003). Quinoprotein alcohol dehydrogenase is involved in catabolic acetate production, while NAD-dependent alcohol dehydrogenase in ethanol assimilation, in *Acetobacter pasteurianus* SKU1108. *J. Biosci. Bioeng.* 96 564–571. 10.1016/S1389-1723(04)70150-4 16233574

[B10] Davidson J. F. Whyte B. Bissinger P. H. Schiestl R. H. (1996). Oxidative stress is involved in heat-induced cell death in *Saccharomyces cerevisiae*. *Proc. Natl. Acad. Sci. U. S. A.* 93 5116–5121. 10.1073/pnas.93.10.5116 8643537 PMC39416

[B11] Dekking F. M. Kraaikamp C. Lopuhaä H. P. Meester L. E. (2005). *A Modern Introduction to Probability and Statistics: Springer Texts in Statistics.* London: Springer-Verlag.

[B12] Dulley J. R. Grieve P. A. (1975). A simple technique for eliminating interference by detergents in the Lowry method of protein determination. *Anal. Biochem*. 64 136–141. 10.1016/0003-2697(75)90415-7 1137083

[B13] Figurski D. H. Helinski D. R. (1979). Replication of an origin-containing derivative of plasmid RK2 dependent on a plasmid function provided in trans. *Proc. Natl. Acad. Sci. U. S. A.* 76 1648–1652. 10.1073/pnas.76.4.1648 377280 PMC383447

[B14] Fukaya M. Tayama K. Tamaki T. Tagami H. Okumura H. Kawamura Y. (1989). Cloning of the membrane-bound aldehyde dehydrogenase gene of *Acetobacter polyoxogenes* and improvement of acetic acid production by use of the cloned gene. *Appl. Environ. Microbiol.* 55 171–176. 10.1128/aem.55.1.171-176.1989 16347820 PMC184073

[B15] Gropp M. Strausz Y. Gross M. Glaser G. (2001). Regulation of *Escherichia coli* RelA requires oligomerization of the C-terminal domain. *J. Bacteriol.* 183 570–579. 10.1128/JB.183.2.570-579.2001 11133950 PMC94912

[B16] Hattori H. Yakushi T. Matsutani M. Moonmangmee D. Toyama H. Adachi O. (2012). High-temperature sorbose fermentation with thermotolerant *Gluconobacterfrateurii* CHM43 and its mutant strain adapted to higher temperature. *Appl. Microbiol. Biotechnol.* 95 1531–1540. 10.1007/s00253-012-4005-4 22434571

[B17] Irving S. E. Corrigan R. M. (2018). Triggering the stringent response: signals responsible for activating (p)ppGpp synthesis in bacteria. *Microbiology* 164 268–276. 10.1099/mic.0.000621 29493495

[B18] Kanchanarach W. Theeragool G. Inoue T. Yakushi T. Adachi O. Matsushita K. (2010). Acetic acid fermentation of *Acetobacter pasteurianus*: relationship between acetic acid resistance and pellicle polysaccharide formation. *Biosci. Biotechnol. Biochem.* 74 1591–1597. 10.1271/bbb.100183 20699583

[B19] Kosaka T. Nakajima Y. Ishii A. Yamashita M. Yoshida S. Murata M. (2019). Capacity for survival in global warming: adaptation of mesophiles to the temperature upper limit. *PLoS One* 14:e0215614. 10.1371/journal.pone.0215614 31063502 PMC6504187

[B20] Kostner D. Peters B. Mientus M. Liebl W. Ehrenreich A. (2013). Importance of *codB*for new *codA*-based markerless gene deletion in *Gluconobacter*strains. *Appl. Microbiol. Biotechnol.* 97 8341–8349. 10.1007/s00253-013-5164-7 23955475

[B21] Langmead B. Salzberg S. L. (2012). Fast gapped-read alignment with Bowtie 2. *Nat. Methods* 9 357–359. 10.1038/nmeth.1923 22388286 PMC3322381

[B22] Lipinska B. Zylicz M. Georgopoulos C. (1990). The HtrA (DegP) protein, essential for *Escherichia coli* survival at high temperatures, is an endopeptidase. *J. Bacteriol.* 172 1791–1797. 10.1128/jb.172.4.1791-1797.1990 2180903 PMC208670

[B23] Matsumoto N. Hattori H. Matsutani M. Matayoshi C. Toyama H. Kataoka N. (2018). A single-nucleotide insertion in a drug transporter gene induces a thermotolerant phenotype of *Gluconobacterfrateurii* by increasing the NADPH/NADP^+^ ratio via metabolic change. *Appl. Environ. Microbiol*. 84:e00354-18. 10.1128/AEM.00354-18 29549098 PMC5930370

[B24] Matsumoto N. Matsutani M. Azuma Y. Kataoka N. Yakushi T. Matsushita K. (2020). In vitro thermal adaptation of mesophilic *Acetobacter pasteurianus* NBRC 3283 generates thermotolerant strains with evolutionary trade-offs. *Biosci. Biotechnol. Biochem*. 84 832–841. 10.1080/09168451.2019.1703638 31851582

[B25] Matsumoto N. Osumi N. Matsutani M. Phathanathavorn T. Kataoka N. Theeragool G. (2021). Thermal adaptation of *acetic acid bacteria* for practical high-temperature vinegar fermentation. *Biosci. Biotechnol. Biochem*. 85 1243–1251. 10.1093/bbb/zbab009 33686416

[B26] Matsushita K. Azuma Y. Kosaka T. Yakushi T. Hoshida H. Akada R. (2016). Genomic analyses of thermotolerant microorganisms used for high-temperature fermentations. *Biosci. Biotechnol. Biochem*. 80 655–668. 10.1080/09168451.2015.1104235 26566045

[B27] Matsutani M. Nishikura M. Saichana N. Hatano T. Masud-Tippayasak U. Theergool G. (2013). Adaptive mutation of *Acetobacter pasteurianus* SKU1108 enhances acetic acid fermentation ability at high temperature. *J. Biotechnol*. 165 109–119. 10.1016/j.jbiotec.2013.03.006 23524057

[B28] McBroom A. J. Johnson A. P. Vemulapalli S. Kuehn M. J. (2006). Outer membrane vesicle production by *Escherichia coli* is independent of membrane instability. *J. Bacteriol.* 188 5385–5392. 10.1128/JB.00498-06 16855227 PMC1540050

[B29] McBroom A. J. Kuehn M. J. (2007). Release of outer membrane vesicles by Gram-negative bacteria is a novel envelope stress response. *Mol. Microbiol.* 63 545–558. 10.1111/j.1365-2958.2006.05522.x 17163978 PMC1868505

[B30] Mechold U. Murphy H. Brown L. Cashel M. (2002). Intramolecular regulation of the opposing (p)ppGpp catalytic activities of Rel(Seq), the Rel/Spo enzyme from *Streptococcus equisimilis*. *J. Bacteriol*. 184 2878–2888. 10.1128/JB.184.11.2878-2888.2002 12003927 PMC135074

[B31] Murata M. Fujimoto H. Nishimura K. Charoensuk K. Nagamitsu H. Raina S. (2011). Molecular strategy for survival at a critical high temperature in *Eschierichia coli*. *PLoS One* 6:e20063. 10.1371/journal.pone.0020063 21695201 PMC3112155

[B32] Nantapong N. Murata R. Trakulnaleamsai S. Kataoka N. Yakushi T. Matsushita K. (2019). The effect of reactive oxygen species (ROS) and ROS-scavenging enzymes, superoxide dismutase and catalase, on the thermotolerant ability of *Corynebacterium glutamicum*. *Appl. Microbiol. Biotechnol.* 103 5355–5366. 10.1007/s00253-019-09848-2 31041469

[B33] Ogino H. Azuma Y. Hosoyama A. Nakazawa H. Matsutani M. Hasegawa A. (2011). Complete genome sequence of NBRC 3288, a unique cellulose-nonproducing strain of *Gluconacetobacterxylinus* isolated from vinegar. *J. Bacteriol.* 193 6997–6998. 10.1128/JB.06158-11 22123756 PMC3232855

[B34] Paul B. J. Ross W. Gaal T. Gourse R. L. (2004). rRNA transcription in *Escherichia coli*. *Ann. Rev. Genetics* 38 749–770. 10.1146/annurev.genet.38.072902.091347 15568992

[B35] Reece K. S. Phillips G. J. (1995). New plasmids carrying antibiotic-resistance cassettes. *Gene* 165 141–142. 10.1016/0378-1119(95)00529-f 7489905

[B36] Robinson M. D. McCarthy D. J. Smyth G. K. (2010). edgeR: a Bioconductor package for differential expression analysis of digital gene expression data. *Bioinformatics* 26 139–140. 10.1093/bioinformatics/btp616 19910308 PMC2796818

[B37] Rodriguez-Verdugo A. Carrillo-Cisneros D. Gonzalez-Gonzalez A. Gaut B. S. Bennett A. F. (2014). Different trade-offs result from alternate genetic adaptations to a common environment. *Proc. Nat. Acad. Sci. U. S. A.* 111 12121–12126. 10.1073/pnas.1406886111 25092325 PMC4143048

[B38] Roghanian M. Semsey S. Løbner-olesen A. Jalalvand F. (2019). (p)ppGpp-mediated stress response induced by defects in outer membrane biogenesis and ATP production promotes survival in*Escherichia coli*. *Sci. Rep.* 9:2934. 10.1038/s41598-019-39371-3 30814571 PMC6393671

[B39] Rudolph B. Gebendorfer K. M. Buchner J. Winter J. (2010). Evolution of *Escherichia coli* for growth at high temperatures. *J. Biol. Chem.* 285 19029–19034. 10.1074/jbc.M110.103374 20406805 PMC2885180

[B40] Schreiber G. Ron E. Z. Glaser G. (1995). ppGpp-mediated regulation of DNA replication and cell division in *Escherichia coli*. *Curr. Microbiol.* 30 27–32. 10.1007/BF00294520 7765879

[B41] Schwechheimer C. Sullivan C. J. Kuehn M. J. (2013). Envelope control of outer membrane vesicle production in Gram-negative bacteria. *Biochemistry* 52 3031–3040. 10.1021/bi400164t 23521754 PMC3731998

[B42] Soemphol W. Deeraksa A. Matsutani M. Yakushi T. Toyama H. Adachi O. (2011). Global analysis of the genes involved in the thermotolerance mechanism of thermotolerant *Acetobacter tropicalis* SKU1100. *Biosci. Biotechnol. Biochem*. 75 1921–1928. 10.1271/bbb.110310 21979075

[B43] Spiess C. Beil A. Ehrmann M. (1999). A temperature-dependent switch from chaperone to protease in a widely conserved heat shock protein. *Cell* 97 339–347. 10.1016/S0092-8674(00)80743-6 10319814

[B44] Stott K. V. Wood S. M. Blair J. A. Nguyen B. T. Herrera A. Perez Mora Y. G. (2015). (p)ppGpp modulates cell size and the initiation of DNA replication in *Caulobacter crescentus* in response to a block in lipid biosynthesis. *Microbiology* 161 553–564. 10.1099/mic.0.000032 25573769 PMC4339654

[B45] Strauch K. L. Johnson K. Beckwith J. (1989). Characterization of *degP*, a gene required for proteolysis in the cell envelope and essential for growth of *Escherichia coli* at high temperature. *J. Bacteriol*. 17 2689–2696. 10.1128/jb.171.5.2689-2696.1989 2540154 PMC209953

[B46] Sun J. Nishiyama T. Shimizu K. Kadota K. (2013). TCC: an R package for comparing tag count data with robust normalization strategies. *BMC Bioinformatics* 14:219. 10.1186/1471-2105-14-219 23837715 PMC3716788

[B47] Taweecheep P. Naloka K. Matsutani M. Yakushi T. Matsushita K. Theeragool G. (2019). In Vitro thermal and ethanol adaptations to improve vinegar fermentation at high temperature of *Komagataeibacteroboediens* MSKU 3. *Appl. Biochem. Biotechnol*. 189 144–159. 10.1007/s12010-019-03003-3 30957194

[B48] Traxier M. F. Summers S. M. Nguyen H.-T. Zacharia V. M. Hightower G. A. Smith J. T. (2008). The global, ppGpp-mediated stringent response to amino acid starvation in *Escherichia coli*. *Mol. Microbiol.* 68 1128–1148. 10.1111/j.1365-2958.2008.06229.x 18430135 PMC3719176

[B49] Trcek J. Jernejc K. Matsushita K. (2007). The highly tolerant acetic acid bacterium *Gluconacetobacter europaeus* adapts to the presence of acetic acid by changes in lipid composition, morphological properties and PQQ-dependent ADH expression. *Extremophiles* 11 627–635. 10.1007/s00792-007-0077-y 17487444

[B50] Vadia S. Tse J. L. Lucena R. Yang Z. Kellogg D. R. Wang J. D. (2017). Fatty acid availability sets cell envelope capacity and dictates microbial cell size. *Curr. Biol.* 27 1757–1767.e5. 10.1016/j.cub.2017.05.076 28602657 PMC5551417

[B51] Varik V. Oliveira S. R. A. Hauryliuk V. Tenson T. (2017). HPLC-based quantification of bacterial housekeeping nucleotides and alarmone messengers ppGpp and pppGpp. *Sci. Rep.* 7:11022. 10.1038/s41598-017-10988-6 28887466 PMC5591245

[B52] Vercruysse M. Fauvart M. Jans A. Beullens S. Braeken K. Cloots L. (2011). Stress response regulators identified through genome-wide transcriptome analysis of the (p)ppGpp-dependent response in *Rhizobium etli*. *Genome Biol.* 12:R17. 10.1186/gb-2011-12-2-r17 21324192 PMC3188799

[B53] Wu Y. Yeh F. L. Mao F. Chapman E. R. (2009). Biophysical characterization of styryl dye-membrane interactions. *Biophys. J.* 97 101–109. 10.1016/j.bpj.2009.04.028 19580748 PMC2711377

[B54] Yang X. Ishiguro E. E. (2003). Temperature-sensitive growth and decreased thermotolerance associated with *relA* mutations in *Escherichia coli*. *J. Bacteriol.* 185 5765–5771. 10.1128/JB.185.19.5765-5771.2003 13129947 PMC193974

