# Supplementary material for: Mutations in degP and spoT Genes Mediate Response to Fermentation Stress in Thermally Adapted Strains of Acetic Acid Bacterium Komagataeibacter medellinensis NBRC 3288
Source: Front Microbiol. 2022 May 12;13:802010. doi: 10.3389/fmicb.2022.802010 (PMC9135448; doi:10.3389/fmicb.2022.802010)
Supplement: Supplementary file 1 [file Data_Sheet_1.docx]

Supplementary Material

## Supplementary Figures

## Fig. S1. Adaptation process of *K. medellinensis* at 34℃.

## The cultivation was performed in 100ml YPGAE medium at 34˚C with a rotary shaking at 200 rpm. When the medium acidity reached to 1.5~2%, the culture was transferred into the new medium as shown with the red arrows. The cultivation was repeated 10 times to get the rapid growing culture, from which ITO-1 strain was isolated.

##

**Fig. S2. Growth comparison among the *gyrB* and *degP* over-expressed strains by dot spot in YPGAE medium.**

Pre-culture were diluted with 0.85% NaCl solution to 10^0^, 10^-1^, 10^-2^, 10^-3^, 10^-4^. The diluted sample (7µL) were dotted on YPGAE plates, and incubated at 30℃, 33℃, and 35℃.

Wild/pP*gyrB*^WT^, and Wild/pP*gyrB*^ITO-1^ are NBRC 3288 strains harboring pP*gyrB*^WT^ and pP*gyrB*^ITO-1^, respectively, and ITO-1/ pP*gyrB*^WT^, and ITO-1/pP*gyrB*^ITO-1^ are ITO-1 strains harboring pP*gyrB*^WT^ and pP*gyrB*^ITO-1^, respectively. pP*gyrB*^WT^ and pP*gyrB*^ITO-1^ are pMV24 having gyrB gene of *K. medellinensis* NBRC 3288 and *K. medellinensis* ITO-1, respectively.

Wild/pMV24, ITO-1/pMV24, and pP*degP*^WT^ and pP*degP*^ITO-1^ are shown in Table S1.


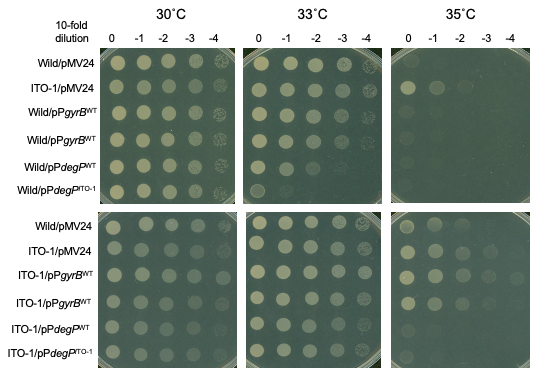


## Fig. S3. Quantification of ppGpp in anion-exchange HPLC column.

## ppGpp analysis was performed basically according to the published methods [Ahmad et al., 2019; Varik et al., 2017], where cell cultures of *E.* *coli* or *Pseudomonas aeruginosa* were filtrated and ppGpp was extracted from the membrane filter by lysis solvent (methanol:acetonitrile:water =2:2:1), and then applied to a MonoQ HPLC column chromatograpy. Briefly, wild, ITO-1, and ΔTGSAct strains were cultivated in YPG or YPGAE medium at 30˚C up to early mid-log phase (Klett unit of ~150, acidity of ~2.0), then rapidly filtrated with PVDF membrane (0.45 mm) under vacuum, washed with small amounts of 0.85% NaCl, and then the washed membrane was put into 8 mL of lysis solvent and sonicated for 2 min. The extracted solvent was diluted with 5mM Tris-HCl (pH 8) 5-fold, and then centrifuged at 9,000 rpm for 10 min to take the supernatant. After filtrated by 0.22 mm PVDF membrane, the supernatant was directly injected into MonoQ 5/50 HPLC column. Samples were eluted at flow rate of 0.5 mL/min. Column was eluted with 5mM Tris-HCl (pH 8) at the first 35 min, then by a linear gradient up to 0.35 M NaCl in 5mM Tris-HCl (pH 8) at the next 35 min, then finally washed with 5mM Tris-HCl (pH 8) containing 0.35 M NaCl at the next 15 min. Elution was monitored at 254 nm.

## References: Ahmad, S., Wang, B., Walker, M. D., Tran, H-K. R., Stogios, P. J., Savchenko, A., Grant, R. A., McArthur, A. G., Laub, M. T., and Whitney, J. C. An interbacterial toxin inhibits target cell growth by synthesizing (p)ppApp. Nature 575, 674–678 (2019) doi.org/10.1038/s41586-019-1735-9

## Varik, V., Oliveira, S. R. A., Hauryliuk, V., and Tenson, T. HPLC-based quantification of bacterial housekeeping nucleotides and alarmone messengers ppGpp and pppGpp. Scientific Reports 7: 11022 (2017) doi:10.1038/s 41598-017-10988-6

## Standard HPLC.

## Each 10 nmol of nucleotides were applied.

1. **HPLC pattern of the extract from the wild strain grown on YPG medium.**

Nucleotides were extracted from 80 ml of the culture, which contained cells of total 17mg of protein. Lower panel showed one of 12.5-fold expanded absorbance from the upper panel. Upper insert shows the retention times and estimated contents (nmols) per cell protein (mg). Lower insert shows the absorption spectrum of the peak of the retention time (red arrow) corresponding to the standard ppGpp. In this case, the absorption does not fit to the absorption of the nucleotide.

1. **HPLC pattern of the extract from ITO-1 grown on YPG medium.**

Sample was extracted from 80 ml of the culture, which contained cells of total 22mg of protein. Panel and insert conditions are the same as described in (B).

1. **HPLC pattern of the extract from ΔTGSAct strain grown on YPG medium.**

Sample was extracted from 80 ml of culture, which contained cells of total 13mg of protein. Panel and inserts conditions are the same as described in (B).

1. **HPLC pattern of the extract from wild strain grown on YPGAE medium.**

Sample was extracted from 220 ml of culture, which contained cells of total 28mg of protein. Lower panel showed 6.25-fold expanded absorbance of the upper panel, different from other data. Inserts are the same as described in (B).

1. **HPLC pattern of the extract from ITO-1 grown on YPGAE medium.**

Sample was extracted from 250 ml of culture, which contained cells of total 20mg of protein. Panel and inserts conditions are the same as described in (B).

1. **HPLC pattern of the extract from ΔTGSAct strain grown on YPGAE medium.**

Sample was extracted from 250 ml of culture, which contained cells of total 22mg of protein. Panel and inserts conditions are the same as described in (B).

**Supplementary Tables**

**Table S1. Strains and plasmids used in this study.**

| Strains and plasmids | Description | Sources & Ref. |
| --- | --- | --- |
| *Komagataeibacter medillinensis* | |  |
| NBRC 3288 | Wild type | Ogino et al., 2011 |
| ITO-1 | Adapted strain of NBRC 3288 at 34℃ | This study |
| ITO-2 | Adapted strain of ITO-1 at 34.5℃ | This study |
| ITO-3 | Adapted strain of ITO-2 at 35℃ | This study |
| Wild/pMV24 | NBRC 3288 harboring pMV24 | This study |
| ITO-1/pMV24 | ITO-1 harboring pMV24 | This study |
| Δ*degP* | NBRC 3288, Δ*degP*::Tc | This study |
| ΔTGSAct | NBRC 3288, SpoT::Tc | This study |
| ΔAct-4 | NBRC 3288, SpoT (Act-4 domain)::Tc | This study |
| Δ*degP*/pMV24 | Δ*degP* harboring pMV24 | This study |
| Δ*degP*/ pP*degP*^WT^ | Δ*degP* harboring pP*degP*^WT^ | This study |
| Δ*degP*/ pP*degP*^ITO-1^ | Δ*degP* harboring pP*degP*^ITO-1^ | This study |
| *Acetobacter pasteurianus* | |  |
| SKU1108 | Wild type | Chinnawirotpisan et al., 2003 |
| TH-3 | Thermally adapted strain of SKU1108 | Matsutani et al., 2013 |
| ΔApAct | SKU1108 with 1.6 kbp region in-frame deletion of APT_00593 | This study |
| *Escherichia coli* |  |  |
| DH5α |  | Grant et al., 1990 |
| Plasmids |  |  |
| pT7Blue | Ap^R^, *lacZα* | Novagen |
| pMV24 |  | Fukaya et al., 1989 |
| pKRP12 | *ori*_MB1_, Ap^R^, Tc^R^ cassette | Reece & Phillips, 1995 |
| pKOS6b |  | Kostnor et al., 2013 |
| pP*degP*^WT^ | pMV24, a putative promoter region of the *adhAB* gene of *K. medellinensis* NBRC 3288, the *degP* gene of *K. medellinensis* NBRC 3288 | This study |
| pP*degP*^ITO-1^ | pMV24, a putative promoter region of the *adhAB* gene of *K. medellinensis* NBRC 3288, the *degP* gene of *K. medellinensis* ITO-1 | This study |
| pT*degP* | pT7Blue, the *degP* gene of *K. medellinensis* NBRC 3288 | This study |
| pΔ*degP* | pT7Blue, the Tc^R^ fragment-inserted *degP* gene of *K. medellinensis* NBRC 3288 | This study |
| pT*spoT* | pT7Blue, the *spoT* gene of *K. medellinensis* NBRC 3288 | This study |
| pΔTGSAct | pT7Blue, the Tc^R^ fragment-inserted *spoT* gene of *K. medellinensis* NBRC 3288 | This study |
| pΔAct-4 | pT7Blue, the Tc^R^ fragment-inserted *spoT* gene of *K. medellinensis* NBRC 3288 | This study |
| pΔApAct | pKOS6b, a fragment for in-frame deletion of the *spoT* gene of *A. pasteurianus* | This study |

**Table S2. Primers used in this study.**

| Primer names | Primer sequences |
| --- | --- |
| 3288-adhpro-Sac(+) | GAGCTCTATTCCGTTCCATCC |
| 3288-adhpro-Xho(-): | CTCGAGAACGTCATTATCCGAAAACGGCAG |
| 3288-DegP-PstXho-F: | CTGCAGCTCGAGTATAACACGGGTCGGCTCTC |
| 3288-DegP-HinXba-R: | AAGCTTCTAGATCATAAAAATGGCCA CGACA |
| 3288-GTPppk-Sal-F: | GTCGACGAAGAACTGGAAGCCTCTGT |
| 3288-GTPppk-Xba-R: | TCTAGATTCACGCGCAGTAAAGACAC |
| 3288-ITO-spoT-5(+): | ATCCGCGTAACCAGAAGATC |
| 3288-ITO-spoT-fsn-5(-): | CACGGTGCCTGACTGCGTTAGCGCCGCCCTGCCGCCCCGC |
| 3288-ITO-spoT-fsn-5(+): | GCGGGGCGGCAGGGCGGCGCTAACGCAGTCAGGCACCGTG |
| 3288-ITO-spoT-3(-): | GTAATCACCCACCTGCAATG |
| 1108-APT0593RI(+): | GAATTCAGGGGTGACGCTACGCTC |
| 1108-APT0593-TGA-Sac(-): | GAGCTCAGCGGCGGCCAGAGCCAC |
| 1108-APT0593-Xba(-): | TCTAGAAAACAGCTACATCACCCC |
| 1108-APT0593-Sac(+): | GAGCTCTGCGCGCGGCCCCCGGC |

**Table S3. Typical data for transcriptome analysis.**

Differentially expressed genes (DEG) were shown in five different combinations. 1) Wild 3288_30˚C, YPGAE/YPG: Comparison of YPGAE grown cells per YPG grown cells of wild strain at 30˚C, 2) ITO-1_30˚C, YPGAE/YPG: Comparison of YPGA and YPG grown cells of ITO-1 at 30˚C, 3) YPG_30˚C, ITO-1/Wild 3288: Comparison of ITO-1 and wild strains grown in YPG at 30˚C, 4) YPGAE_30˚C, ITO-1/Wild 3288: Comparison of ITO-1 and wild strains grown in YPGAE at 30˚C, 5) ITO-1_YPGAE, 34˚C/30˚C: Comparison of ITO-1 strain grown in YPGAE at 30˚C and 34˚C.

DEG is shown as log2[FC] (log2[Fold change]) with FDR (False discovery rate) that should be less than 0.05.

**(A) Stress Response Genes**

**
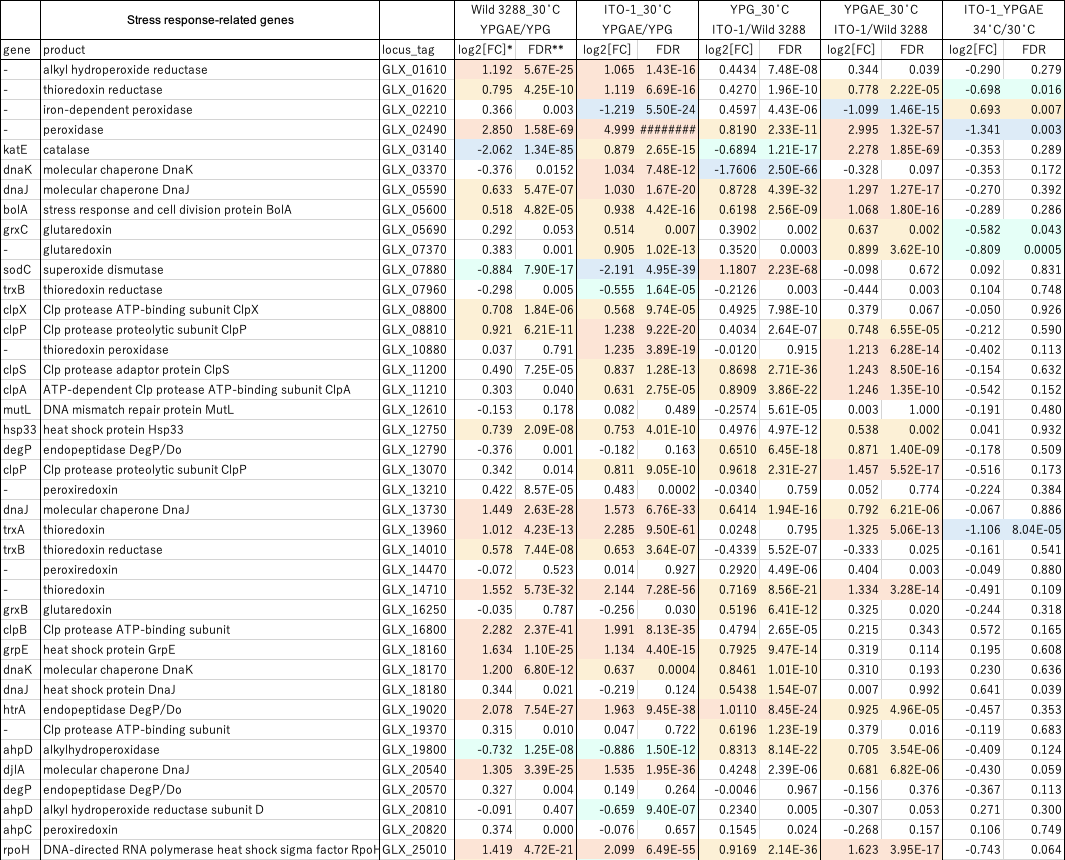
**

**
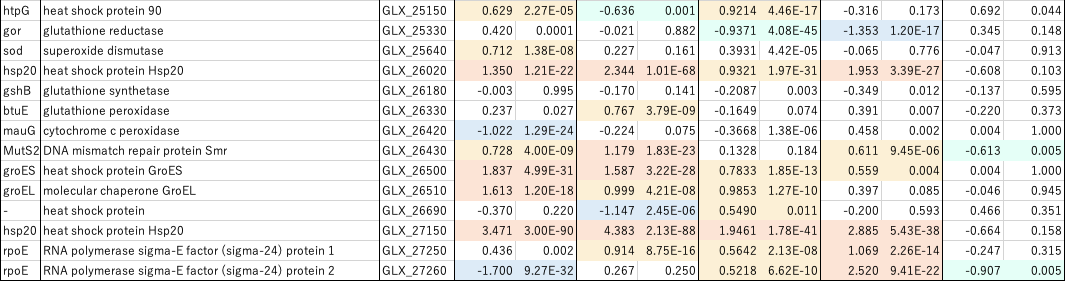
**

**(B) Translation related Genes**

**
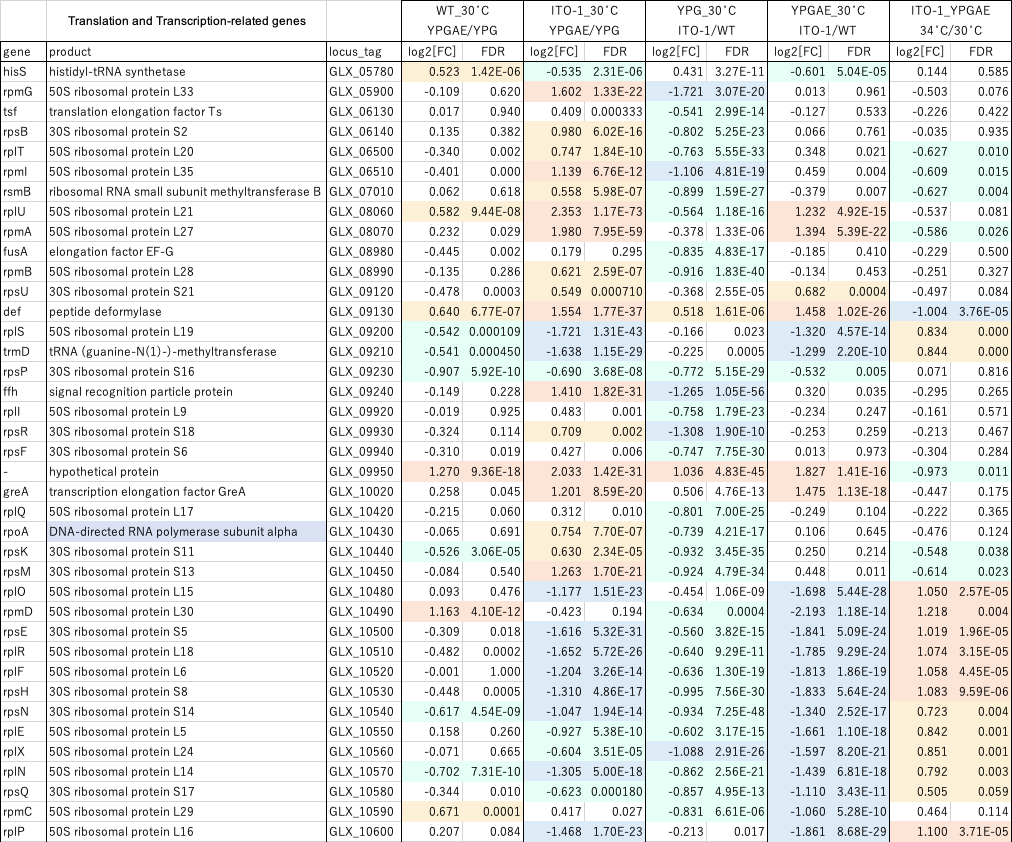
**

**
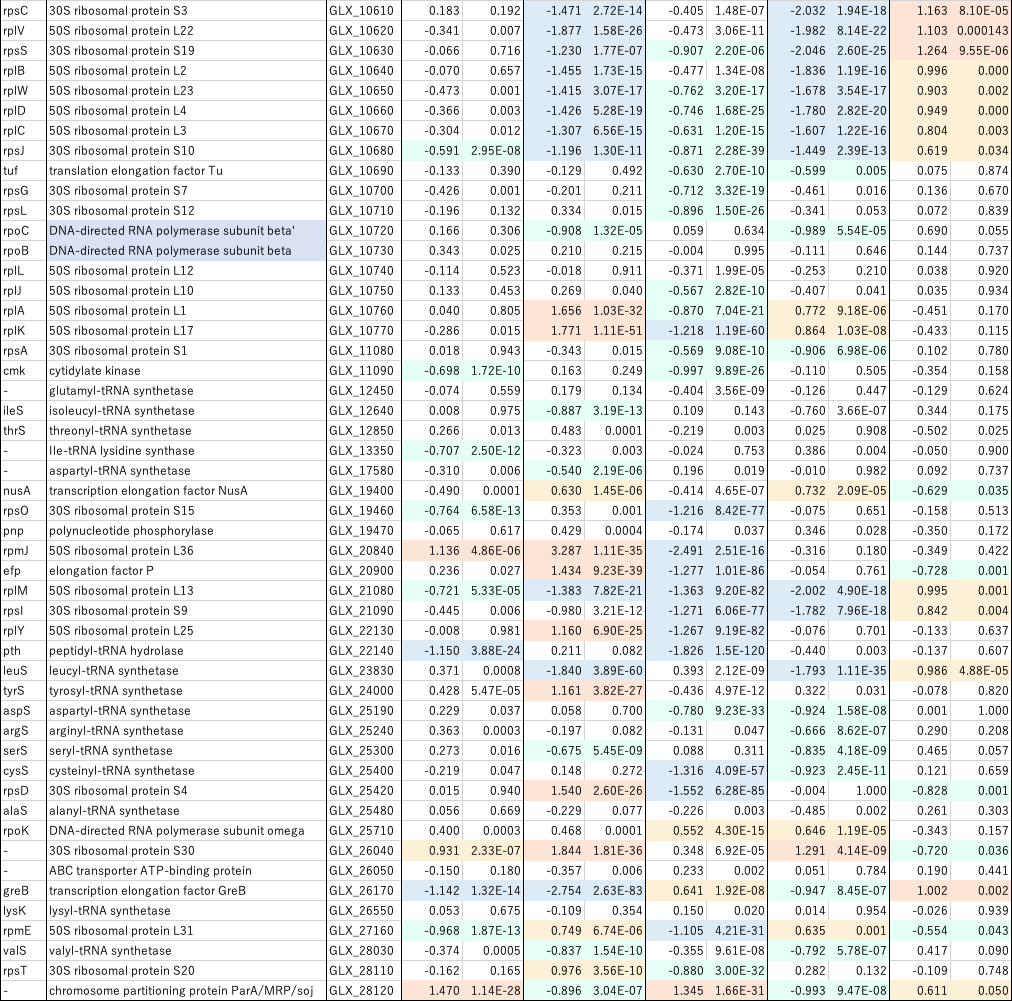
**

**(C) Energy metabolism related Genes**

**
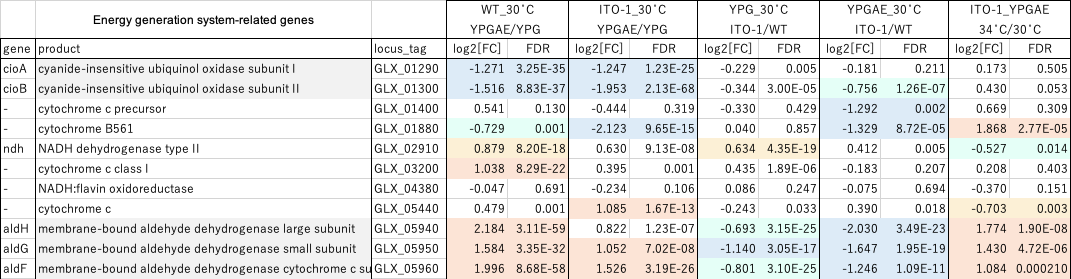
**

**
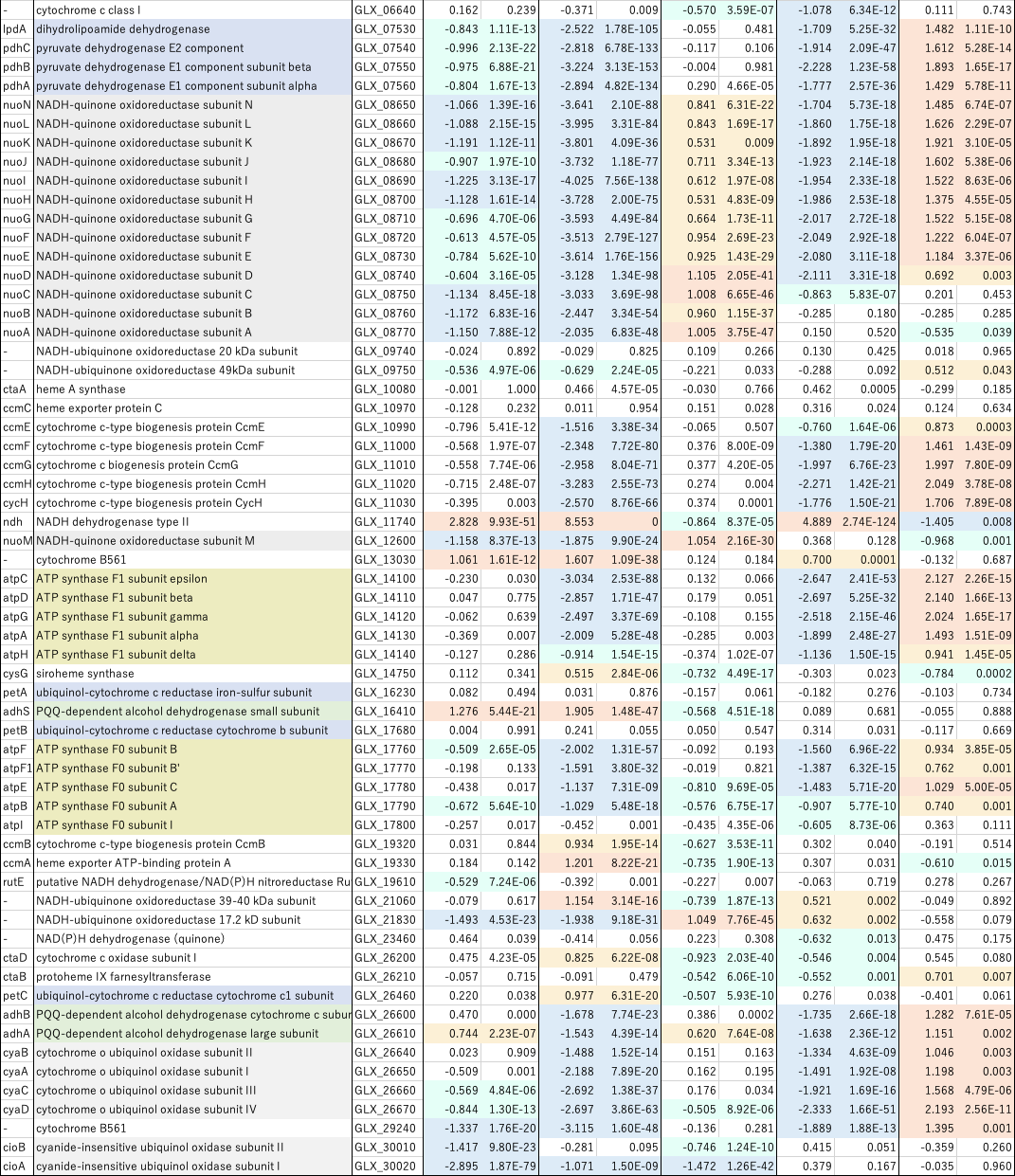
**
